# Enhancing the therapeutic efficacy of nanoparticles for cancer treatment using versatile targeted strategies

**DOI:** 10.1186/s13045-022-01320-5

**Published:** 2022-09-12

**Authors:** Hailong Tian, Tingting Zhang, Siyuan Qin, Zhao Huang, Li Zhou, Jiayan Shi, Edouard C. Nice, Na Xie, Canhua Huang, Zhisen Shen

**Affiliations:** 1 Department of Otorhinolaryngology and Head and Neck Surgery, The Affiliated Lihuili Hospital, Ningbo University, 315040 Ningbo, Zhejiang China; 2 State Key Laboratory of Biotherapy and Cancer Center, West China Hospital, Sichuan University, and Collaborative Innovation Center for Biotherapy, Chengdu, 610041 China; 3 Department of Biochemistry and Molecular Biology, Monash University, Clayton, 3800 VIC Australia; 4 West China School of Basic Medical Sciences and Forensic Medicine, Sichuan university, Chengdu, 610041 China

**Keywords:** Drug delivery, Targeted strategies, Active targeting, Stimuli-responsive materials, Cancer treatment

## Abstract

Poor targeting of therapeutics leading to severe adverse effects on normal tissues is considered one of the obstacles in cancer therapy. To help overcome this, nanoscale drug delivery systems have provided an alternative avenue for improving the therapeutic potential of various agents and bioactive molecules through the enhanced permeability and retention (EPR) effect. Nanosystems with cancer-targeted ligands can achieve effective delivery to the tumor cells utilizing cell surface-specific receptors, the tumor vasculature and antigens with high accuracy and affinity. Additionally, stimuli-responsive nanoplatforms have also been considered as a promising and effective targeting strategy against tumors, as these nanoplatforms maintain their stealth feature under normal conditions, but upon homing in on cancerous lesions or their microenvironment, are responsive and release their cargoes. In this review, we comprehensively summarize the field of active targeting drug delivery systems and a number of stimuli-responsive release studies in the context of emerging nanoplatform development, and also discuss how this knowledge can contribute to further improvements in clinical practice.

## Background

Cancer is one of the leading causes of death worldwide, and despite the current arsenal of anticancer strategies, the number of patients is continuously increasing [1, 2]. Statistics have shown that one in 6 women and one in 5 men worldwide develop a tumor in their lifetime [3, 4] which accounts for nearly 1 in 6 deaths. The main reason behind the poor treatment efficacy is the low targeting ratio of therapeutics which can also induce severe side effects on healthy tissues [5, 6]. Therefore, there is an urgent need for site-specific delivery of therapeutic agents to the tumor region. For this reason, nanotechnology-based formulations have been the focus of a large body of research as effective approaches for overcoming the bottlenecks of undirected biodistribution, undesired side effects and high-dose administration [7].

**Fig. 1:**
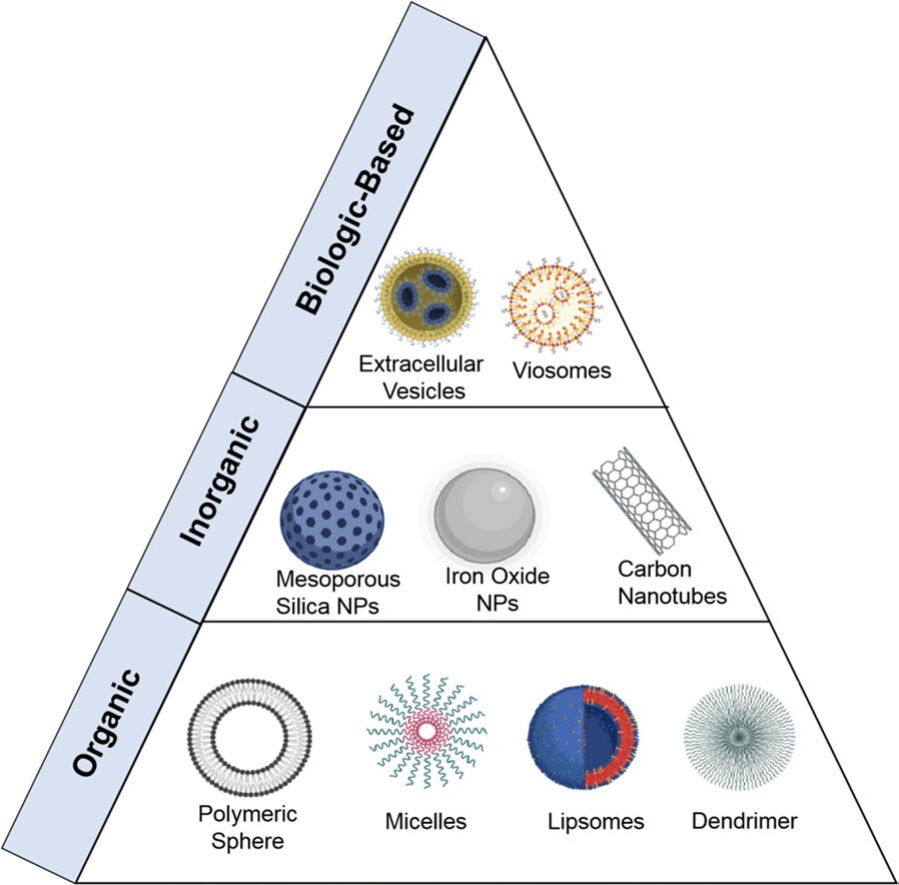
The main drug delivery systems in tumor treatment

With the increased uptake in nanomedicine, various versatile nanoformulations with excellent biocompatibility and pharmacokinetic properties, such as micelles, liposomes, nanoparticles, and nanoemulsions, have exhibited great potential for the delivery of novel anti-cancer drugs (Fig. 1) [8–10]. These nanoparticles can effectively address the poor water solubility and undesired adverse effects often observed during the delivery of therapeutic agents and prolong their blood circulation time for enhanced tumor accumulation, thereby markedly facilitating their use as therapeutic agents for tumor therapies [10–12]. Importantly, these novel nanomedicines generated by encapsulating specific therapeutic agents in nanocarriers can achieve satisfactory tumor targeting by utilizing the EPR effect-mediated passive targeting strategy [13, 14]. Furthermore, active targeting can also be effectively achieved by conjugating nanomedicines with ligands that can specifically target overexpressed receptors on the tumor cells [15–17]. The inclusion of active targeting ligands over the surface of nanoparticles improves their targeting toward tumor cells (on-targets) rather than healthy cells (off-targets). Therefore, this feature of ligands not only increases the therapeutic index but also minimizes the associated side effects.

Recently, stimuli-responsive nanoparticles have also been proposed as a promising active targeting strategy for tumor treatment [18–22]. Specifically, an acidic environment, high levels of reactive oxygen species (ROS) and glutathione (GSH), and overexpression of specific enzymes in the tumor microenvironment (TME) can contribute to the development of stimuli-responsive nanoparticles for targeted drug delivery, as these nanoparticles maintain their stealth features in the normal physiological environment but upon homing to targeted sites or the local microenvironment are responsive and release encapsulated agents [18–21]. Moreover, functionalized nanoparticles can also be activated by external stimuli including magnetic fields, light, and ultrasound, to realize efficient tumor accumulation and controlled drug release in a temporal and spatial-specific fashion [22]. It should be noted that these stimuli-responsive nanoparticles also overcome many of the disadvantages of conventional nanoagents by site-specific tumor targeting and controlled drug release, such as providing improved therapeutic agent delivery, overcoming the off-target side effects and enhancing the therapeutic benefits.

For these reasons, smart targeting nanoparticles for efficient tumor accumulation and controlled release of therapeutic agents are gaining widespread attention as personalized treatment regimens [23]. This review focuses on important recent advances in versatile targeting strategies for tumor treatment, including receptor-mediated and stimuli-responsive targeting nanoparticles, which present exceptional potential as multimodal delivery platforms against cancer. Special emphasis has been given to stimuli-responsive nanoparticles as novel targeting strategies and their potential to support paradigm changes in cancer treatment. Furthermore, the current challenges and future prospects of receptor-mediated and stimuli-responsive targeting nanoparticles are also discussed.

## Receptor-mediated active targeting strategy

**Fig. 2:**
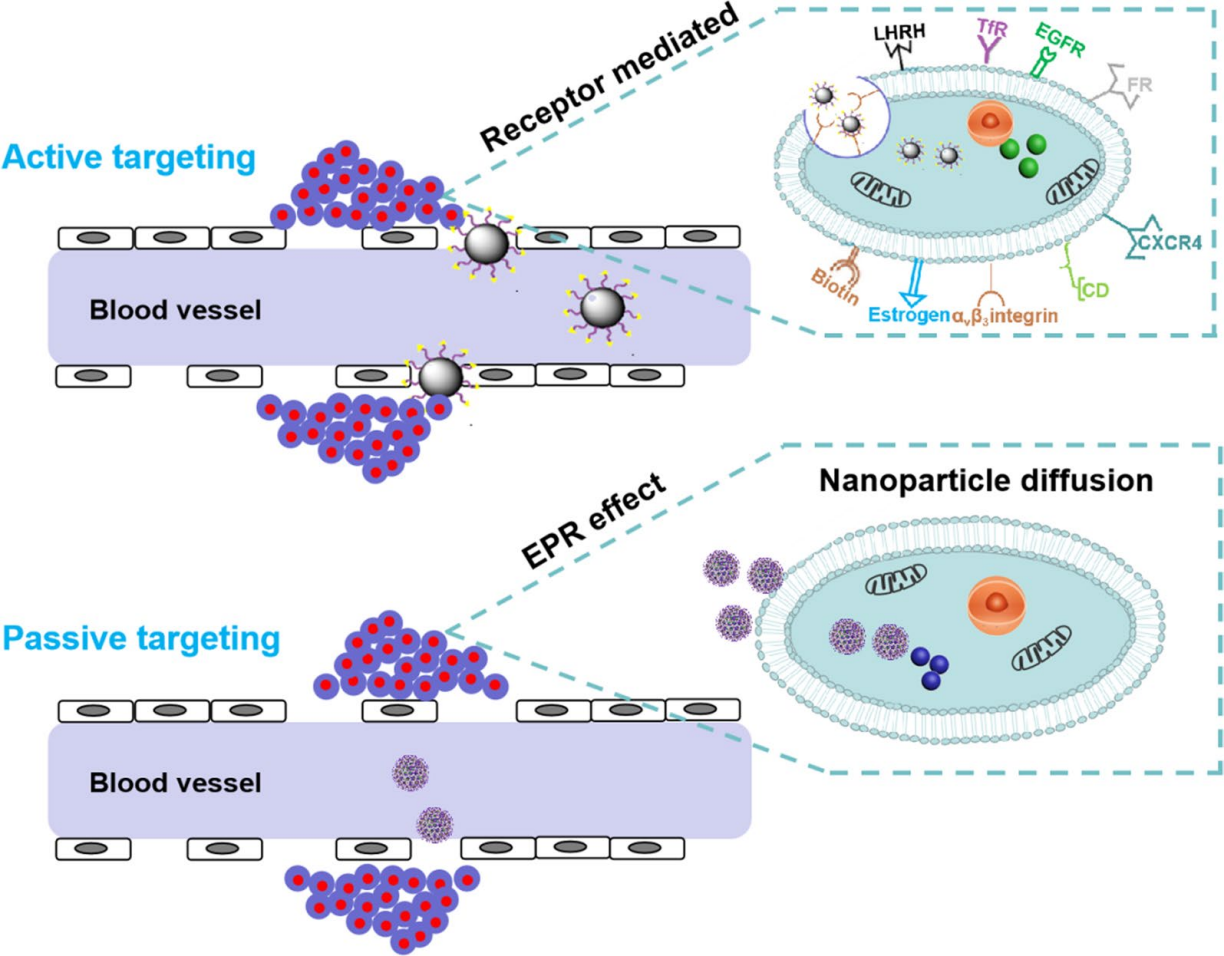
Schematic representation of receptor-mediated active targeting and passive targeting through the EPR effect

Nanoparticles can be used to overcome the TME barriers and deliver pharmaceutical active ingredients to the tumor sites by either passive or active targeting strategies (Fig. 2). Passive targeting involves the transport of nanoparticles through the leaky tumor vasculature-mediated EPR effect, leading to nonspecific tumor accumulation. In active targeting strategies, specialized chemical moieties or ligands can be conjugated to the surface of nanoparticles and are capable of site-specific delivery to tumor sites. Generally, these ligands are chosen based upon expression levels of specific receptors and their internalization at the target site. It should be noted that these receptors or cell surface markers should be overexpressed on target cells, facilitating the homing action of nanoparticles. Additionally, stimuli-responsive nanoparticles have also been considered a promising active targeting strategy for tumor treatment, as they enable the safe delivery of the agents while controlling their release at the target sites.

**Fig. 3:**
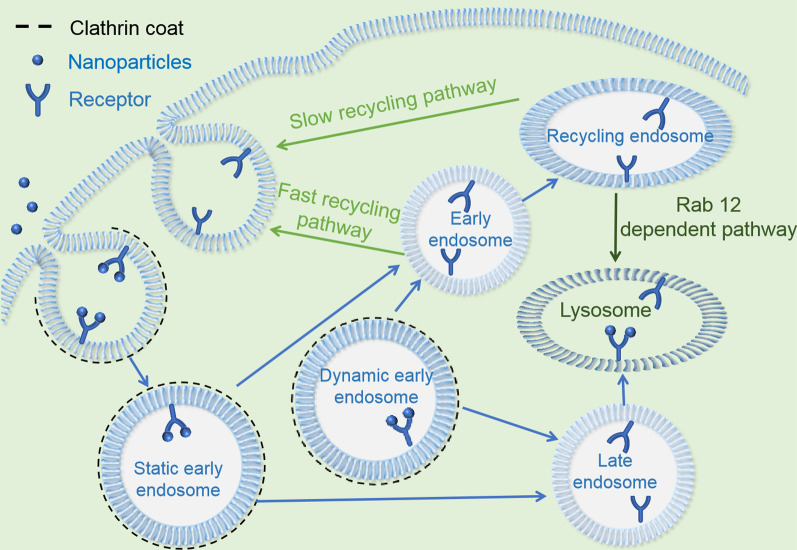
Schematic representation of receptor-mediated endocytosis

A number of receptors are overexpressed on the tumor cell surface, which enables them to be distinguished from healthy cells at the molecular level. Moreover, the progressive use of tumor proteomics and bioinformatics has contributed significantly to the discovery of these specific receptors [24]. The addition of specific ligands on the nanoplatform surfaces allows them to selectively target tumor cells. Once bound to specific receptors, the encapsulated therapeutic agent nanoplatform can be effectively taken up into tumor cells through receptor-dependent endocytosis (Fig. 3). Therefore, strategies for targeting drugs to tumor cell surface receptors to enhance tumor accumulation have attracted extensive attention in recent years. Table 1 summarizes some of the specific receptors overexpressed on various tumor cells along with their related ligands. Utilizing cell surface active targeting strategies has greatly advanced tumor treatment. Some of these approaches are summarized in the following sections.

**Table 1:** The overexpressed receptors on various tumor cells and their ligands

Receptor	Ligands	Tumor	Refs.
Folate	Folic acid	Breast, lung, cervical cancer, hepatocellular carcinoma	[414–419]
CD14	anti-CD14 mAb	Prostatic cancer	[420]
CD22	anti-CD22 mAb	Lymphoma cancer	[421, 422]
CD44	HA, chondroitin sulfate	Breast, Melanoma	[423–426]
αvβ3 integrin	RGD peptide	Endothelial, glioma, lung, melanoma, breast cancer	[427–434]
Transferrin	TfR ligand, transferrin	Breast cancer, Glioblastoma	[435–438]
HER2	Trastuzumab Breast anti-HER2 scFv neu peptide (FCDGFYACYADV) KCCYSL (P6.1 peptide)	Breast cancer	[439–441]
Estrogen	Estrone, 17 β-Estradiol, tamoxifen	Breast cancer	[442–446]
Chemokine (CXCR4)	LFC131 peptide, anti-CXCR4 mAb, Peptide R, Peptide T22	Breast, lung cancer, hepatocellular carcinoma, Lymphoma	[447–454]
LHRH	Peptide	Breast cancer	[106, 107]
Biotin	Biotin	Breast, lung, cervical cancer, hepatocellular carcinoma	[455–460]
PSMA	A10 PSMA Apt, anti-PSMA	Prostatic cancer	[461–463]
VEGF	anti-VEGF mAb	Pancreatic cancer	[464, 465]
IL4	AP1 peptide	Colon, glioblastoma	[466–468]
IL4	Pep-1	Lung cancer	[469–471]
IL13	IL13 peptide	Glioblastoma	[472–474]
Asialoglycoprotein receptor (ASGPR)	Lactobionic acid, galactose	Hepatocellular carcinoma	[475, 476]

### Epidermal growth factor receptor (EGFRs)-based active targeting

The EGFR, a transmembrane protein, is involved in the occurrence of several types of cancers, including lung, pancreatic, colorectal, and breast cancers [24]. Activation of the EGFR is triggered by the binding of ligands, including EGF, transforming growth factor-α (TGF-α), epiregulin, heparin-binding EGF, betacellulin, amphiregulin, and neuregulin G2β. This enables protein kinase (PK) to transfer a phosphate molecule from adenosine triphosphate (ATP) to the tyrosine residues, resulting in phosphorylation of the intracellular domain, which mediates a signaling cascade pathway [25, 26]. Eventually, this process can result in tumorigenesis and cancer progression, thereby making EGFR one of the main anticancer targets [27, 28]. The most commonly used EGFR targeting agents in clinical use are monoclonal antibodies (mAbs) and small molecule tyrosine kinase inhibitors (TKIs). The mAbs can be directly applied to deliver therapeutic agents to tumor cells through drug-Ab complexes or modified on the surface of the nanoplatform-loaded therapeutic agents [29, 30].

Recently, EGFR-based nanoplatforms have been widely explored against cancers [31–35]. These nanoparticles are generally internalized into the cells through an EGFR-mediated endocytosis process, resulting in the formation of lysosomes and release of encapsulated drugs for cancer treatment. As an exemplar, Nan and co-workers prepared versatile nanoplatforms capable of specific codelivery of DOX and cisplatin to tumor sites by utilizing an EGFR-targeted approach [33]. These targeted nanoparticles showed high stability with sustained cargo release, showing satisfactory killing effects in lung cancer models. In a similar approach, Liang et al. prepared versatile nanoplatforms functionalized with anti-EGFR Ab for lesion-specific delivery of carmustine to malignant glioblastomas for growth suppression [34]. Confirming the role of the EGFR, Shuai and co-workers reported higher internalization of an anti-EGFR monoclonal antibody-conjugated nanoplatform in EGFR-positive human skin squamous cell carcinoma compared to EGFR-negative breast cancer [35]. Furthermore, Choi and co-workers demonstrated that binding EGFR-targeting Abs to gemcitabine (Gem) encapsulated nanoplatforms could effectively inhibit tumor growth [36]. Gupta and colleagues constructed a Gem encapsulated nanoplatform against pancreatic cancer through covalent binding to EGFR antibodies [37], presenting higher cytotoxicity of the designed nanoplatform for EGFR-overexpressing pancreatic cell lines. Moreover, anti-EGFR functionalized Fe_2_O_3_ nanoparticles can be used as magnetic resonance imaging contrast agents for tumor diagnosis [38, 39]. In an interesting review article, Yi and colleagues discussed the role of EGFR tyrosine kinase inhibitors in targeted nanoplatforms for tumor treatment [40].

Supported by the rapid advancement of nanomedicine, these inhibitor-loaded nanoparticles are showing improved bioavailability, prolonged blood circulation, enhanced tumor accumulation and reduced off-target side effects, leading to significant augmentation of therapeutic efficacy [41, 42] supporting their continued development.

### αvβ3 integrin receptor-mediated active targeting

Integrin receptors, consisting primarily of transmembrane glycoproteins, can mediate cell–cell and cell-extracellular matrix adhesion [43]. More than 23 integrin heterodimers have been identified in humans to date [44]. These receptors control the connection between the extracellular matrix (ECM) and the cell cytoskeleton as well as maintaining communications between cells [43, 45]. The extracellular domains of integrins have strong affinity for the proteins (collagen, fibronectin, laminin and vitronectin) in the ECM. Furthermore, integrins can play a significant part in several signaling pathways involved in cell proliferation after combining with the ECM [46]. It is possible to target integrin receptor-positive tumor cells through functionalized nanoparticles containing an integrin targeting motif (such as RGD-containing peptides and polymers). This approach has been extensively explored [47–53]. For example, Lu and co-workers prepared cyclic RGD peptide-functionalized nanoplatforms for paclitaxel (PTX) delivery to glioblastoma cells overexpressing αvβ3, resulting in antitumor effects in in vivo models [49]. In another example, Li et al. prepared RGD-conjugated resveratrol loaded human serum albumin nanoparticles, which showed higher internalization efficiency (approximately 3.6-fold higher) as well as improved tumor suppression features compared to the non-functionalized formulation [50]. Amreddy and co-workers developed RGD-functionalized nanoparticles for the delivery of therapeutic agents (PTX and cisplatin) to αvβ3 integrin receptor-overexpressing lung cancer cells and found that the RGD-targeted nanoformulations showed higher endocytosis efficiency (approximately 1.4-fold higher) compared with non-RGD-functionalized formulations [51]. Pan and co-workers developed RGD-modified fluorescent nanoplatforms for simultaneous fluorescence-guided and targeted delivery of epirubicin to overexpressed αvβ3 integrin in esophageal cancer, resulting in the designed nanoplatforms not only reducing epirubicin-induced cardiotoxicity but also improving the therapeutic effect in comparison to free agents [52]. Recently, Roy et al. prepared pH-responsive nanoparticles for the effective delivery of raloxifene to breast cancer cells through RGD-modified nanocarriers. The designed nanoparticles showed good cytotoxicity and antitumor efficacy toward α_v_β_3_ positive breast cancer cells and a 4T1-bearing mouse model [53]. In another recent study, Wang’s group reported a bispecific assembling peptide antiCD3-G7-RGD for tumor immunotherapy [54]. The RGD was used to improve tumor accumulation and cell internalization via the integrin receptor-mediated endocytosis process. The anti-CD3 was designed to target the CD3 receptor on T lymphocytes and induce a T cell-mediated immune response against tumor cells overexpressing integrin αvβ3, resulting in satisfactory antitumor effects. In summary, nanoparticles can preferentially and effectively target integrin binding sites in tumors (e.g., the RGD motif), thereby providing a solid basis for developing precision tumor treatment strategies [55].

### Folate receptor (FR)-mediated active targeting

FRs, a class of glycoproteins, have been classified into three subtypes namely FRα, FRβ and FRγ. It should be noted that FRα and FRβ can closely bind to the tumor cell membrane via a glycosylphosphatidylinositol anchor, while FRγ has only been reported in hematopoietic cells [4, 56–58]. Among them, FRα is the most widely generated FR subtype and is overgenerated in various tumor cells, especially in breast, lung, kidney, cervical, and ovarian cancer [59–61]. Moreover, FR can transport folate into tumor cells via the receptor-mediated endocytosis process [62]. For this reason, a number of FA-based nanoplatforms have been prepared for increased internalization of therapeutic agents by tumor cells [63–65]. In one example, Murgia et al. prepared an organic/inorganic hybrid nanoplatform modified by FA-chitosan conjugates to load upconverting NaYF_4_ nanoparticles and daunorubicin for tumor therapy [62]. The FA modification significantly improved the cellular uptake of the nanoparticles, and an in vivo xenograft model also showed a positive antitumor effect. In another example, Wang et al. designed an FA-conjugated chitosan loaded rutin prepared palladium nanoplatform for FA-mediated targeting treatment. The introduction of FA into the designed nanoplatform significantly improved the endocytosis efficiency of the nanoparticles in breast cancer cells. The designed nanoplatform was shown to considerably suppress cell proliferation as evidenced by a cell viability assay [66]. Mechanistically, FRs can identify and bind to extracellular FA-modified nanoparticles and then transport them into the tumor cells through a FR-mediated endocytosis process [67]. In these nanoparticles, the FA portion is used as a tumor-targeting ligand. On binding to the FR on tumor cells, the cell membrane can invaginate and pinch off to form endosomes which subsequently reach lysosomes or other organelles. The drug-encapsulated nanoparticles can dissociate from the FR and effectively release the encapsulated drug at the TME for tumor treatment.

### Transferrin (Tf) receptor-mediated active targeting

As the critical Fe^3+^ pool in the body, Tf plays an important role in Fe metabolism and delivery. To meet the growing requirements of Fe for maintaining cell growth and division, transferrin receptors (TfR) are frequently overexpressed on the surface of a number of tumors including pancreas, breast, prostate, colon, and lung cancer, with high affinity to Tf [68–71]. This has prompted scientists to use the TfR as an active targeting site in the design of novel anti-cancer delivery platforms. TfR can be employed either for Tf-mediated targeting and internalization of therapeutic agents or to block normal receptor function, resulting in cell death [72–74]. In an interesting recent article, Zhang et al. developed a novel transferrin protein corona (Tpc)-modified CuGd nanoplatform (Tpc-CuGd) for tumor-targeting photothermal and chemodynamic synergistic therapy [75].

In summary, various Tf-modified nanoparticles have been developed for the targeted delivery of therapeutic agents to tumor sites, which can preferentially deliver therapeutics into TfR-overexpressing tumor cells by receptor-mediated internalization [76], showing excellent antitumor effects with few side effects.

### Human epidermal growth factor receptor 2 (HER2)-mediated active targeting

The HER family, comprising HER1, HER2, HER3, and HER4, plays a crucial part in the pathogenesis of various tumors including gastric and breast cancer [77, 78]. HER-targeting-based strategies may address tumor chemoresistance as their associated receptors usually possess tyrosine kinase catalytic activity [79]. Among these, the HER2 receptor is commonly studied in breast cancer as it is overexpressed > 20% of patients [80]. While the HER2 receptor does not have a natural ligand, it can dimerize with other ErbB family receptors, which results in activation of the HER signaling pathways [79]. A significant challenge in developing targeted drugs has been the identification and preparation of HER2-specific artificial ligands with specificity and colloidal stability. Recently, a variety of monoclonal antibodies (Abs) and their fragments, as well as some peptide drugs, have been integrated as targeting units on nanoparticles against HER2 overexpressing cancer. The introduction of trastuzumab (TZ), a humanized anti-HER2 Ab, endows nanoparticles with excellent therapeutic efficacy for breast cancer treatment [81]. It can block cell cycle arrest and reduce angiogenesis by disturbing downstream HER2 signaling activity. The interaction between TZ and HER blocks receptor cleavage and activates the response of Ab-dependent cellular cytotoxicity and receptor degradation following internalization of the TZ-HER2 complex. Pertuzumab (PZ), another humanized mAb, has been used to suppress heregulin-mediated activation of HER2 phosphorylation and tumor proliferation [82]. Nanoparticles functionalized with anti-HER2 Abs or its fragments can be effectively used for specific delivery of therapeutic agents to HER2-overexpressed tumor cells by the HER2 receptor-mediated endocytosis process [83] which enhances therapeutic efficacy with fewer side effects.

### Estrogen receptor-mediated active targeting

Estrogen is a steroid hormone that plays a critical part in maintaining reproductive system function, bone homeostasis, brain development, and cardiovascular remodeling [84]. Among the three forms (estrone (E1), estradiol (E2), and estriol (E3)), E2 is the crucial for the progression of breast, endometrial, and ovarian cancers [85, 86]. Estrogen function relies primarily on its binding and subsequent activation of two structurally different estrogen receptors (ERα and ERβ) [87]. Therefore, these related receptors are considered members of the nuclear receptor superfamily.

It has been reported that following intracellular uptake of estrogen-modified nanoparticles by receptor-mediated endocytosis, intracellular ERs can carry these nanoparticles toward the nucleus for nuclear targeting [88]. Furthermore, these receptors have been found overexpressed on several tumor cell surfaces. In a recent application, Kapara and co-workers [89] reported a straightforward and non-destructive 3D surface-enhanced Raman spectroscopy (SERS) imaging strategy to track the cellular internalization of AuNPs modified with an anti-ERα Ab in MCF-7 cells. It was found that these modified nanoparticles were effectively internalized by tumor cells using the ERα receptor-mediated endocytosis process for enhanced tumor treatment.

### Cluster of differentiation (CD) receptor-mediated active targeting

The CD receptor family comprises surface receptors mainly present on cancer stem cells (CSCs), including CD14, CD22, CD36, CD44, and CD133, which can be used as promising delivery targets against tumor metastasis. Among them, CD44, a transmembrane adhesion glycoprotein, has been commonly used to target receptors for targeted tumor treatment [90–92]. Hyaluronic acid (HA), a ligand with good biocompatibility, has been widely used in CD44 receptor-mediated active targeting delivery systems. It can be readily obtained due to its abundance as a natural polymer compared with polymers that require multiple step chemical synthesis [93, 94]. HA-functionalized nanoplatforms can effectively deliver therapeutic agents to tumor cells through CD44 receptor-mediated active targeting, with an excellent cytotoxic profile and tumor kill. For example, Kim et al. [94] reported a HA modified, trio-stimuli receptive and on-demand triggerable nanoplatform for multimodal cancer treatment. These HA-enveloped nanoparticles effectively suppressed tumor growth in comparison to groups without HA modification. In general, HA is modified on the surface of nanoparticles to specifically bind to CD44 receptors that are overexpressed in tumor cells, thus mediating tumor endocytosis. In addition, HA has the tendency to be degraded to smaller fragments in the presence of hyaluronidase which is also abundantly present in the TME [95]. The versatile characteristics of HA as a targeted and enzyme-responsive ligand make it a promising candidate for application in specific drug delivery systems.

### Other receptor-mediated active targeting systems

In addition to the receptors mentioned above, other receptors have also been used to design targeted anti-cancer nanoplatforms, including chemokine, biotin, and luteinizing hormone-releasing hormone (LHRH) receptors [96–100]. For example, chemokine receptor type 4 (CXCR4) is a class of G-protein-coupled receptor that plays an important part in tumor metastasis by gathering tumor cells along chemokine gradients. Several peptide-functionalized nanoplatforms have been prepared for targeting CXCR4 receptor-positive cancers. For example, Albericio et al. developed circular peptide T22-functionalized mesoporous silica for the effective delivery of chemotherapeutic agents to tumor cells [101]. Wang and co-workers prepared epirubicin-encapsulated polymeric nanoparticles that clearly improved therapeutic efficacy in hepatocellular carcinoma by conjugating the LFC131 peptide to increase the affinity [102]. Similarly, Murakami’s group also developed cellulose nanoparticles with the LFC131 peptide for targeted tumor treatment [103]. Xiao et al. designed a novel nanoplatform to target CXCR-4 to effectively induce p53 expression in hepatocellular carcinoma models. Combining the CXCR4-targeted p53 mRNA nanoplatform with anti-PD-1 treatment effectively induced cellular reprogramming and immune components of the tumor microenvironment in established hepatocellular carcinoma models [104]. It should be noted that the suppression of chemokine signaling can modulate the normal immune function in epithelial cells because CXCR4 plays a significant role in normal cell growth and angiogenesis [105]. There is there an urgent need to design novel chemokine inhibitors that do not disturb the function of healthy cells. A number of LHRH receptors have been found in breast, ovarian and prostate cancer, but their expression is low or absent in the corresponding healthy tissues [106, 107]. Therefore, several nanoplatforms modified with LHRH peptides have been explored for the targeted delivery of therapeutic agents [108–111]. For instance, LHRH peptide conjugated nanoparticles prepared by Tang and co-workers enhanced cellular uptake and tumor suppression in comparison to the non-LHRH targeted formulations [112]. Moreover, Taheri and co-workers designed LHRH peptide-functionalized methotrexate-encapsulated nanoparticles with higher therapeutic efficacy against cancer [113]. In addition, Zhang’s group reported the anti-cancer ability of LHRH receptor-targeted mitoxantrone-encapsulated versatile nanoplatforms in vivo, demonstrating augmented tumor suppression with the targeted liposomes in comparison to non-targeted formulations [111]. Although these receptor-mediated strategies have shown potential advantages for drug delivery, several factors, such as ligand stability, orientation, and density, must be taken into consideration to preserve the function of the targeting ligand.

## Stimuli-responsive targeting strategies

**Fig. 4:**
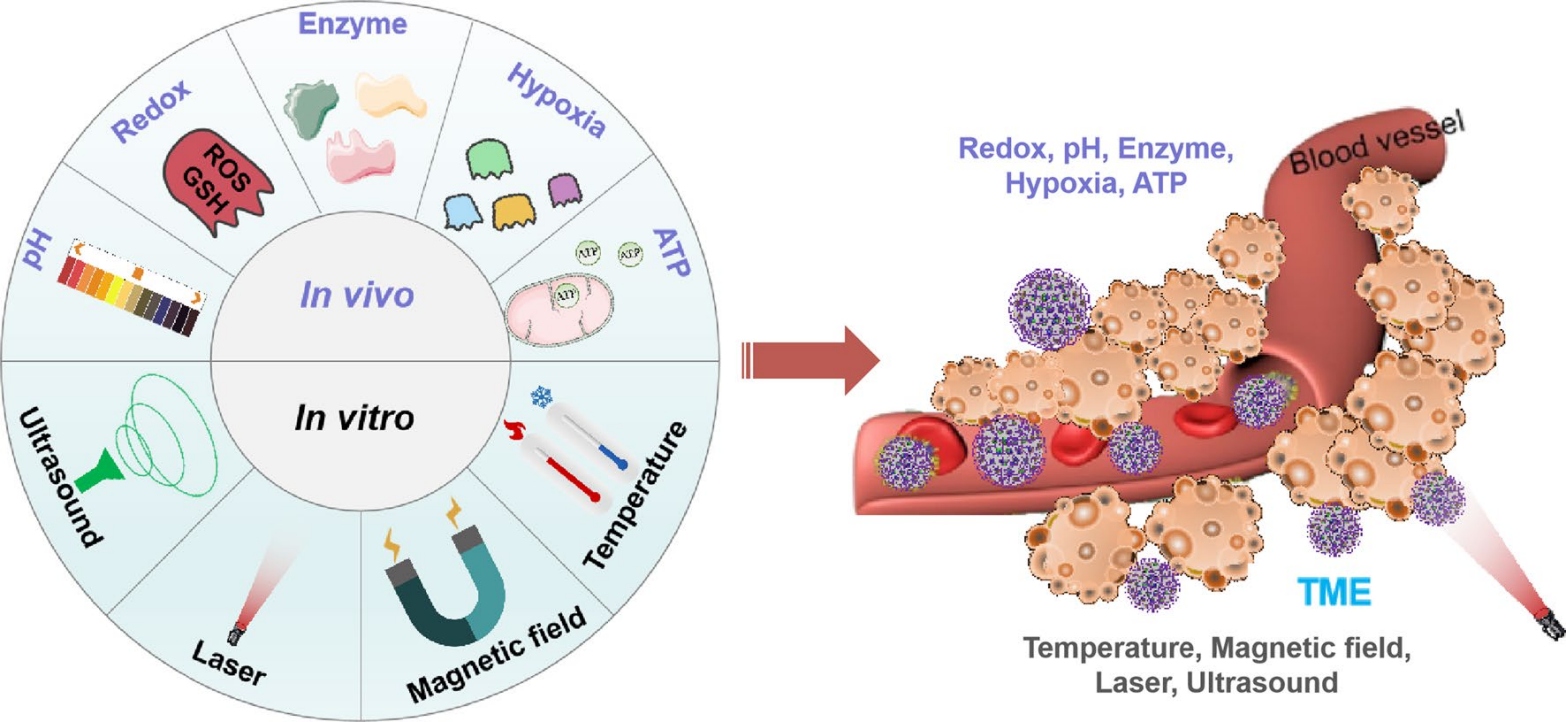
Schematic representation of exogenous and endogenous stimuli-responsive nanoplatforms for tumor therapy

Unique features of the TME include an acidic environment, a high concentration of GSH and ROS, and increased expression of specific enzymes (MMP-2/cathepsin B). Therefore, nanoparticles incorporating TME-responsive components can pave the way for targeted drug delivery and tumor treatment. In response to these endogenous stimuli, alterations in molecular function and dispersion behavior, morphology, and degradation kinetics can be induced. This facilitates either intracellular internalization or escape from endosome/lysosomal degradation and release of pharmaceutical active ingredients [114]. In addition to endogenous responsive nanosystems, some exogenous stimuli-responsive nanoparticles also show beneficial targeting behavior by utilizing controllable external factors, such as lasers, temperature, ultrasound, and magnetism. Several examples of endogenous and exogenous responsive nanoplatforms are presented below (Fig. 4).

### Endogenous Stimuli-responsive targeting strategies

#### Redox-responsive targeting strategies

**Fig. 5:**
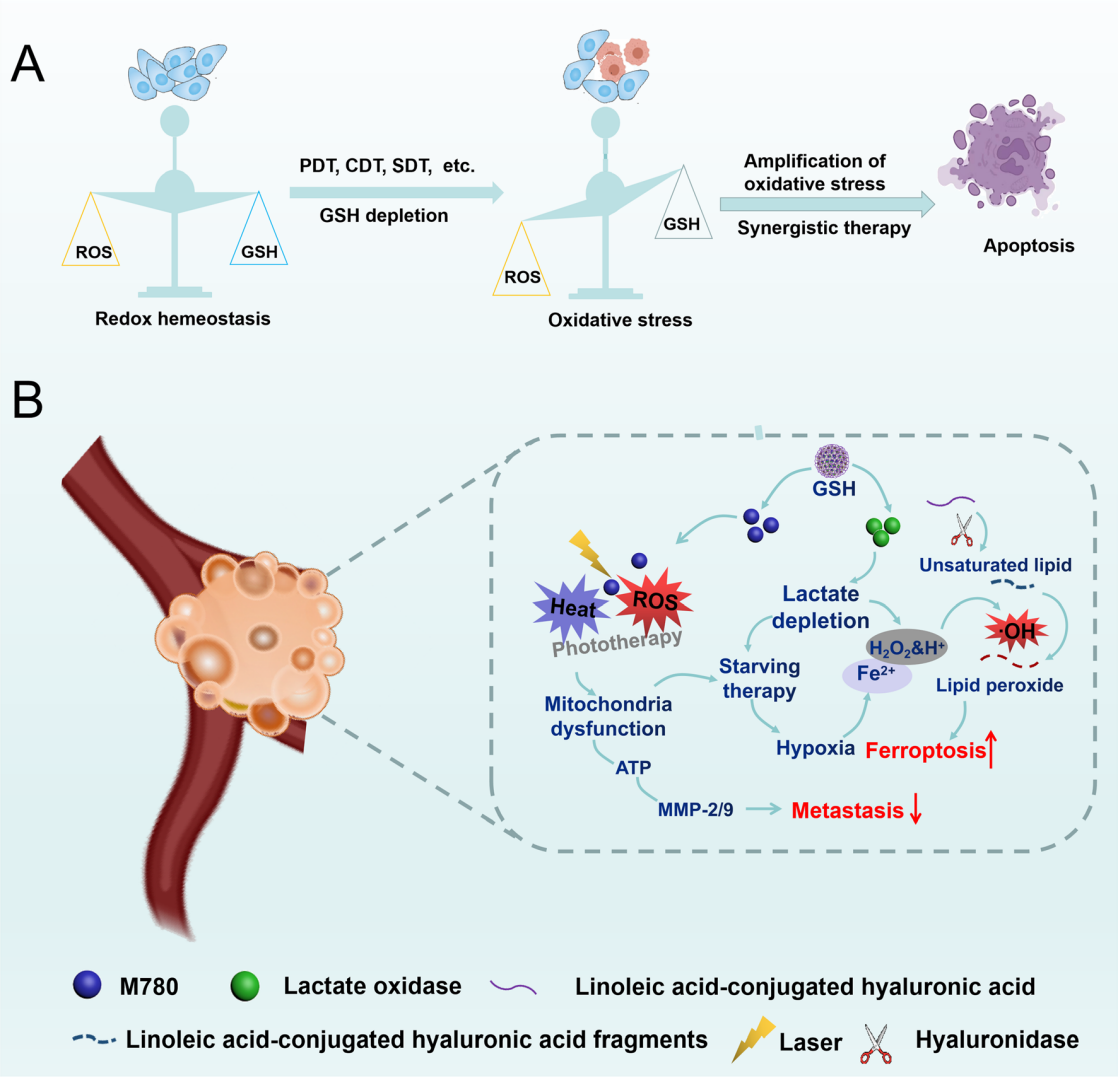
(**A**) Schematic illustration of amplified oxidative stress based on intracellular ROS for incurring tumor cell apoptosis. **B** Schematic illustration of the anti-metastasis performance of the GSH-responsive nanoplatforms

Redox species in tumor cells form a complex antioxidant defense system to modulate redox homeostasis, playing an important role in the cell life cycle [115]. Common reactive oxygen species (ROS) include hydroxyl radicals (·OH), singlet oxygen (^1^O_2_), and hydrogen peroxide (H_2_O_2_). It should be noted that H_2_O_2_ is a stable and nontoxic ROS, while others have a short half-life and can be effectively transformed into powerful toxic reagents [116, 117]. On the one hand, H_2_O_2_ can be used as a substrate for O_2_ production with the aid of a specific enzyme to alleviate tumor microenvironment hypoxia in some O_2_-demanding therapeutic strategies [118]. On the other hand, the H_2_O_2_ can also be converted into other highly active ROS, including ^1^O_2_, O_2_·^−^ and ·OH [116]. This increased ROS can result in oxidative stress, such as lipid peroxidation (LPO), and protein and DNA impairment [119]. In addition, glutathione (GSH), as an antioxidant, is commonly distributed in tumor cells at concentrations up to 2–10 mM, playing a significant role in consuming ROS and modulating redox homeostasis [120, 121]. Furthermore, a high GSH concentration can make tumor cells resistant to various treatments [122]. Therefore, it is advantageous to develop redox-sensitive nanoparticles for the delivery of therapeutic agents to trigger treatments such as chemodynamic therapy (CDT) (Fig. 5). In addition, to further improve the therapeutic profile, ROS generation combined with GSH depletion can effectively disturb redox homeostasis to augment oxidative stress, thus resulting in tumor cell apoptosis [123]. ROS are generated by the partial reduction of O_2_ which is necessary for maintaining the normal function of aerobic organisms using energy provided from four electron reduction reaction [124–129]. As shown in Table 2, most efforts have been to develop ROS-responsive building blocks, which can be combined with chemotherapeutics to achieve excellent antitumor activity with few side effects.

**Table 2:** ROS-responsive building blocks for cancer treatment

Type of chemical bond	Nanoplatform	Tumor model	Therapy strategies	Refs.
Thioketal linker	Polyprodrug NP_DOX/Cy_	Breast cancer	Chemotherapy	[493]
Phenylboronate ester	G5.NHAc-Toy@TF nanocomplexes	Breast cancer	Chemotherapy, CDT	[494]
	pPBA(TL)-MN	Breast cancer	Immunotherapy	[495]
Bilirubin	Dox@bt-BRNPs	Cervical carcinoma	Chemotherapy	[496]
	TH-302@BR-Chitosan NPs	Cervical carcinoma	Chemotherapy PTT	[497]
Gallic acid-ferrous nanocomplex	BSO/GA–Fe(II)@ liposome	Breast cancer	CDT	[119]
Ru nanoparticle	HA-Ru NAs	Breast cancer	PTT, PDT, CDT	[498]
FePt nanoparticle	FePt/MoS2-FA nanocomposites	Breast cancer	Immunotherapy, PTT	[499]
Manganese ferrite nanoparticle (MFN)	MFMSN-Ce6	Melanoma	SDT	[500]
Horseradish peroxidase	Lipo@HRP&ABTS	Breast cancer	PTT	[501]
	PEG-TiO_1+x_ NRs	Breast cancer	SDT, CDT	[502]
Catalase	CAT@Pt (IV)-liposome	Breast cancer	Chemotherapy, RT	[133]
	CAT@HA-HMME NPs	Colorectal cancer	SDT	[503]
Bis(3,4,6-trichloro-2-(pentyloxycarbonyl) phenyl) oxalate	POCL	Cervical carcinoma	PDT	[504]

##### ROS-responsive targeting strategies

Hypoxia, which can cause tumorigenesis and cancer progression, has been considered as a significant biomarker in cancer theranostics and targeted treatment. Moreover, it is controlled by the overgeneration of VEGF and hypoxia induced factor (HIF-1α) in tumor cells [130], resulting in decreased sensitivity of cancers to radiotherapy (RT), causing chemoresistance and also greatly affecting the efficacy of O_2_-related treatments, such as photodynamic therapy (PDT) and sonodynamic therapy (SDT) [131]. Recently, researchers have developed versatile ROS-responsive nanoparticles through catalase (CAT)-mediated tumor site-specific O_2_ generation to alleviate hypoxia for enhancing tumor treatment [132]. For instance, Zhang et al. developed liposomes loaded with a cisplatin-prodrug functionalized phospholipid and CAT [133], alleviating the chemoresistance caused by hypoxia. Further, the liposome encapsulation also endowed the prepared nanoplatforms with satisfactory biocompatibility and a high tumor accumulation profile. Treatment with the designed liposomes induced the highest level of DNA impairment in tumor cells exposed to X-rays in comparison to the control group. In addition, a range of nanocarriers with CAT mimicking activity, including MOF, MnO_2_, CeO_2_, Pt, and Pd [131, 134–138], have also shown greatly potential in nanomedical applications. It should be noted that MnO_2_ is well known to convert H_2_O_2_ into O_2_ under the action of the acidic TME with the disruption of MnO_2_-based nanoparticles [138, 139]. These ROS-responsive nanoparticles capable of stimulating tumor site-specific O_2_ production provide a practical strategy for improving the sensitivity of RT and chemotherapy.

Hydroxyl radicals (·OH) are not only an important component of ROS, but also the main product of the Fenton reaction for tumor-targeted therapy [140]. Moreover, the unique characteristics of slight acidity and overproduction of H_2_O_2_ in the TME offer a suitable environment and reactants for the Fenton reaction compared to normal cells. Tang et al. reported on the use of chemodynamic therapy (CDT using Fenton or Fenton-like reactions for ·OH-producing tumor treatment [141]. Another promising application of endogenous H_2_O_2_ in the TME is to activate CDT for specific cancer treatment [142]. To achieve this, a number of H_2_O_2_-sensitive nanoparticles have been developed, and many efforts have also been devoted to replenishing H_2_O_2_ in tumor cells [143]. Among these strategies, iron-based nanoparticles have been widely applied to generate highly toxic ·OH for tumor treatment. As the intratumoral H_2_O_2_ concentration (50 – 100 Μm) is not sufficient to generate adequate amounts of ·OH, Gao et al. prepared Au-Fe_3_O_4_-based nanoparticles for nanocatalytic cancer treatment. In this nanosystem, Au first catalyzes intracellular glucose oxidation into gluconic acid and H_2_O_2_. The Fe_3_O_4_-triggered Fenton reaction then converts H_2_O_2_ into ·OH radicals inducing tumor cell death [144]. Both in vitro and in vivo results confirmed that the designed nanoparticles presented a satisfactory tumor inhibition ratio. Many other iron-free Fenton nanocatalysts including transition metal-based, precious-metal-based, sulfide-based nanocatalysts and their composites multifunctional radical therapeutics have been developed [145].

Recently, Chen and colleagues prepared copper peroxide (Cu_2_O_2_) nanoparticles with the features of reversible degradation to generate self-supplying H_2_O_2_ through changes in Ph [146]. The H_2_O_2_ could be effectively catalyzed by Cu^2+^ to generate highly toxic ·OH. These nanoparticles showed improved tumor inhibition efficacy in comparison to the controls. Additionally, H_2_O_2_ has also been used for NO-based gas treatment. This has been called a “green” treatment approach for cancer therapy, as it shows minimal toxicity for normal tissues while offering metabolic benefits that are not achievable through chemotherapy or other traditional therapeutic modalities [147]. For example, Chen and colleagues developed mesoporous silica nanoparticles as biocompatible nanovehicles for the delivery of arginine and glucose oxidase (Gox) [148]. These nanoparticles used encapsulated Gox to provide a degrading glucose reaction to increase intracellular H_2_O_2_ concentration, which can then oxidize arginine into NO under the action of specific NO synthase. As the levels of glucose increase, the tumor microenvironment became more acidic, allowing H_2_O_2_ to facilitate the NO production. After treatment with the designed nanoparticles, tumor volumes were considerably reduced and the mice had longer survival times. In another mode, H_2_O_2_ can be used as a stimulus for the disruption of nanoparticles, leading to controllable release. For instance, a novel oxidation-sensitive polymeric carrier has been used to prepare antitumors nanoplatforms [149]. Among them, poly (propylene sulfide) as a hydrophobic block promotes H_2_O_2_-sensitive transformation from insoluble to soluble forms. Poly (propylene sulfide) conjugated with PEG can rapidly self-assemble into nanoparticles and decompose upon confrontation with H_2_O_2_, suggesting great promise as a delivery platform. Overall, nanoparticles containing H_2_O_2_-sensitive groups are expected to become more widely used in stimuli-triggered disintegration and specific cancer treatment [150, 151].

##### Reactive nitrogen species (RNS)-responsive targeting strategies

NO, the first gas molecule for therapy, has attracted attention because of its excellent diffusivity and cell membrane penetration, endowing it with broad biological activities and therapeutic potential [152–158]. It has been reported that matrix metalloproteinases (MMPs), which comprise a family of enzymes that can degrade matrix proteins, are capable of depletion of collagen through activation of NO, resulting in improved penetration ability of the prepared nanoparticles [159, 160]. In addition, NO can react with ^1^O_2_ to generate highly toxic peroxynitrite (ONOO^−^) which has a stronger tumor cell killing ability [161]. ONOO- can convert pro-MMPs into MMPs to degrade the extracellular matrix to enhance the penetration ability of nanoparticles and induce DNA impairment. NO and ONOO- can cause mitochondrial dysfunction by reducing mitochondrial membrane potential and inhibiting the generation of ATP, which effectively suppresses ATP-related tumor-derived vesicles and tumor metastasis [162]. Moreover, the derived RNS and superoxide can effectively kill cancer cells by inducing nitrosative or oxidative stress, DNA or mitochondrial impairment and improving inflammatory reactions, resulting in accelerated cell apoptosis [147, 163–165]. However, there are still some concerns regarding the delivery of NO by different nanomaterials due to difficulties in obtaining efficient encapsulation and precise release [166]. To overcome the drawbacks of the current NO delivery carriers and NO donors, an interesting approach was reported to transport NO for tumor treatment using prodrug self-assembling nanoplatforms of NO donors. Briefly, phenylsulfonylfuroxan was used as the NO donor in the synthesis of a prodrug using ester and disulfide bonds. The insertion of disulfide bonds facilitates the self-assembly of polymers in solution. Subsequently, the multiresponsive tumor-targeting NO nanoparticles can be obtained by adding FA onto the surface, which can achieve the effective delivery of NO to tumor regions, leading to accurate NO release and inducing tumor cell apoptosis [167]. Researchers have also developed other NO donors, such as Roussin’s black salt, metal NO complexes, and S-nitrosothiols [168–171].

NO may also relieve hypoxia in the tumor area through vasodilation, which promotes PDT efficacy [172], further improving the combined effects of PDT and NO in cancer therapy. To improve penetration into tumor tissue in PDT-mediated tumor treatment, researchers typically combine rare-earth up-conversion nanomaterials with different photosensitive therapeutic agents [173, 174]. However, this poses new risks in the preparation and biosecurity of such nanoparticles. The combination of ROS and RNS responsive strategies into the same nanoparticles with good biological safety can be expected to provide an efficient and all-in-one anticancer treatment.

##### GSH-responsive targeting strategies

A number of nanocarriers comprised of disulfide bonds, carbon-diselenide bonds, diselenide bonds, or a sulfonyl group [175–182] have been prepared by cross-linking reactions. Overgenerated GSH can effectively break various disulfide bonds, thus causing disintegration of nanoparticles and accurate cargo release in cancer cells. It should be noted that nanoplatforms with disulfide bonds embedded in mesoporous silica nanoparticles show fast biodegradation and are emerging as promising nanovehicles [183].

For the development of GSH-sensitive nanoplatforms, the co-assembly of amphipathic block copolymers and therapeutic agents with GSH-responsive groups into several nanosystems (such as liposomes, nanoparticles, and micelles) has been considered as a potential application approach [184]. Nanoplatforms bearing GSH-cleavable prodrugs have also been developed, which can be effectively modulated to toxic therapeutic agents by excessive intracellular GSH [185–188]. For example, Sun et al. loaded a trimeric prodrug into FA functionalized polylactic-coglycolic acid hybrid nanoparticles, where the chemotherapeutic camptothecin (CPT) was conjugated to NIR croconaine dyes through disulfide bonds [189]. This novel nanoprodrug had a high CPT loading efficiency and exhibited rapid drug release when exposed to GSH. Additionally, the photothermal effect of the cocaine dyes further facilitated disulfide linker cleavage. The encapsulated croconaine dyes endowed this nanoparticle with NIR fluorescence and photoacoustic imaging properties for tumor treatment.

Platinum drugs (e.g., cisplatin, carboplatin and oxaliplatin) currently remain the most commonly used chemotherapeutic agents against a number of tumors [190, 191]. However, there are numerous problems with these drugs in clinical use, such as lack of specificity and severe side effects on normal organs. Therefore is a growing tendency to develop prodrug-based nontoxic Pt(IV)s that can be converted into highly toxic Pt(II) through the reduction of GSH [192]. Farokhzad’s group developed self-assembled nanoparticles comprised of PEGlipid and Pt (IV) prodrug for tumor treatment [193]. On one hand, this nanoscale strategy facilitated the delivery of cargoes across cell membranes into cells by endocytosis. On another other, these prodrugs had a GSH-depleting feature, resulting in the release of Pt(II) to act on DNA and trigger tumor cell apoptosis.

In addition to the above GSH-responsive nanoparticles, multivalent metal ions such as Fe^2+^ and Fe^3+^ Cu^+^ and Cu^2+^ and Mn^2+^ and Mn^4+^also show GSH-responsive behavior due to a shift in valency, [194]. These reduced metal ions can be further applied for diagnosis or improved treatment. In one example, ultrasmall (4 nm) cerium oxide nanoparticles (CeO_2_ NPs) were rapidly etched, leading to the opening of nanochannels in the mesoporous silicon nanoparticles when exposed to vitamin C or GSH, resulting in controlled antitumor drug release [195]. Recently, our group prepared versatile Cu-MOF nanoparticles loaded with VK3 for enhanced CDT by regulating GSH and H_2_O_2_ in the tumor microenvironment [196, 197]. Cu^+^ and Cu^2+^ showed better catalytic capability than classical Fe-dependent Fenton agents. The satisfactory antitumor effects presented by these Cu-based nanoparticles, and the cascade-enhanced chemo-chemodynamic therapy approach provide an opportunity for the application of such novel nanoplatforms for HCC treatment. Furthermore, future advancements, such as improved targeting, can effectively improve the efficacy and use of such approaches, which should be beneficial to cancer treatment [196, 197]. Additionally, the consumption of GSH plays an important role in metal-based chemodynamic therapy. For instance, Liu et al. developed advanced metal-based nanoparticles through chemodynamics for multimodal tumor treatment [198]. GSH acted on the designed nanoparticles and effectively reduced Mn^4+^, Mn^3+^, and Cu^2+^ into Mn^2+^ and Cu^+^, accompanied by GSH consumption. Inductively coupled plasma optical emission spectrometry (ICP-OES) was used to support the rapid release of Cu and Mn from the nanoparticles in an acidic environment containing GSH. The nanoparticles displayed specific recognition and homotypic targeting profiles to MCF-7 cells. Combining metal ions with GSH-consumption in the TME could become a more promising targeted strategy for CDT

##### ROS and GSH dual-responsive tumor-targeting strategy

As mentioned above, intracellular redox regulation has been considered an effective strategy against cancer. However, the ROS produced from the catalytic oxidation of H_2_O_2_ can be removed by the overgenerated GSH, compromising therapeutic interventions. To overcome this, selective enhancement of oxidative stress through depleting GSH levels and simultaneously elevating ROS concentrations can be a specific and promising strategy in cancer treatment [199]. For instance, Liang and colleagues designed an oxidative stress-amplified nanoplatform for disturbing mitochondrial redox balance, which comprised atomically dispersed Au anchored onto a carbon-dot surface modified with cinnamaldehyde and triphenylphosphine [200]. The acidity of endosomes facilitates the dissociation of cinnamaldehyde. Subsequently, the nanoparticles rapidly react with GSH, accompanied by ROS generation, resulting in the elevation of ROS and the simultaneous reduction of GSH. As a result, levels of mitochondrial GSH in tumor cells were obviously decreased after incubation with the prepared nanoparticles. In addition, the prepared nanoparticles with enhanced oxidative stress possessed excellent anticancer effects against HepG-2 tumors. These groups of designed nanoparticles also showed prolonged survival times and few side effects against various tumor models, which can be attributed to the fact that normal tissues, unlike the TME, do not have high redox levels.

#### pH-responsive targeting strategies

**Fig. 6:**
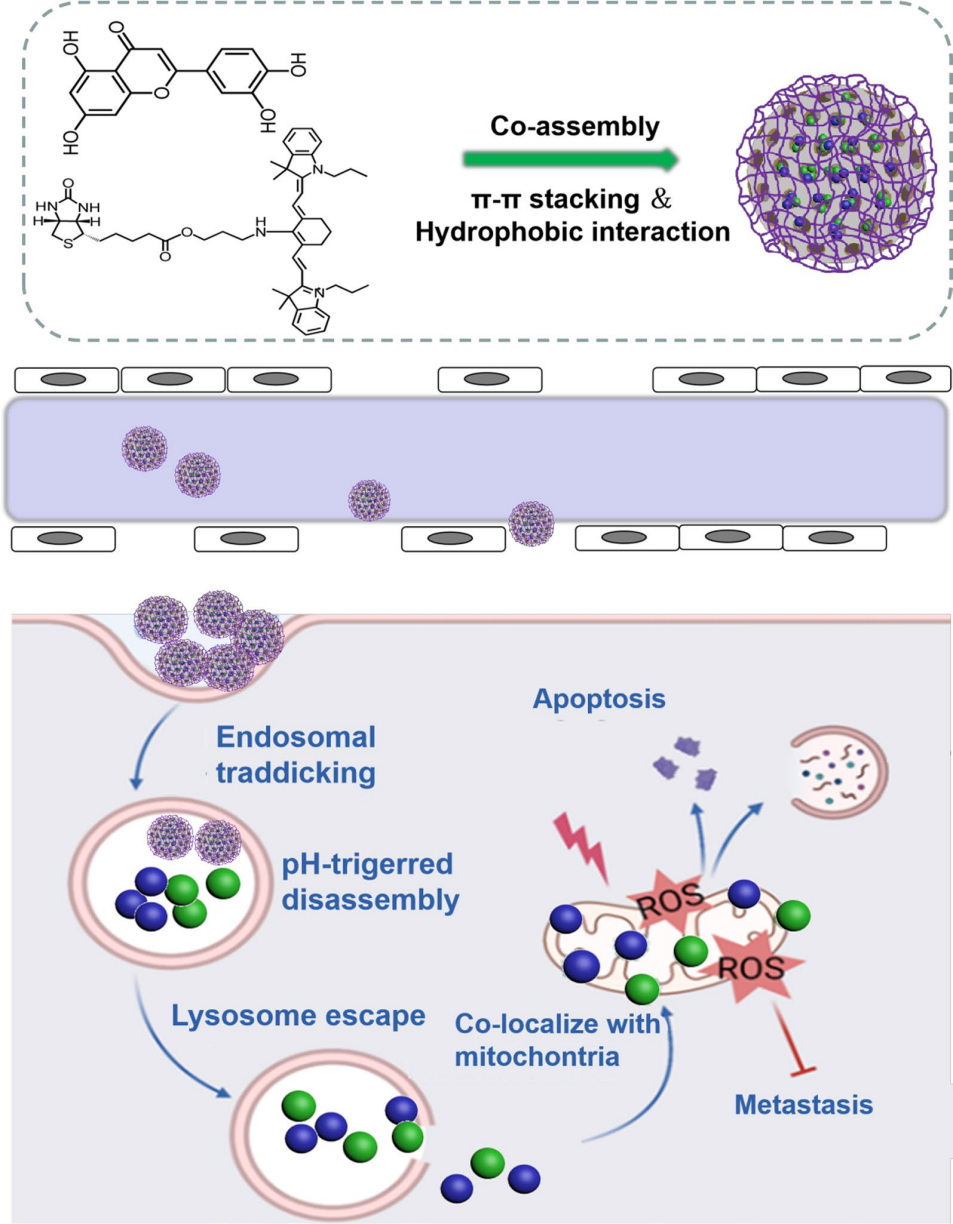
Schematic illustration of pH-responsive nanoplatforms for the delivery of therapeutic agents. The nanoplatforms can effectively accumulate in the tumor sites via the EPR effect. In the tumor microenvironment, acidic conditions can effectively trigger drug release for tumor treatment

pH-sensitive nanocarriers have been extensively explored to design versatile nanoplatforms for targeted drug delivery. The TME usually has a lower extracellular pH (pHex) with a mean value of ~ 6.5 in comparison to healthy tissue [201]. Generally, compared with healthy cells, tumor cells rapidly consume glucose for glycolysis with rapid lactate production to obtain the energy required for maintaining their proliferation regardless of oxygen content; consequently, the higher metabolism rate of tumor cells has been recognized as a major cause of the acidic TME [202]. Additionally, tumor cell, lysosomes and endosomes also have a lower pH (endosomal pH (pHen)) in comparison to pHex [203, 204]. Therefore, pH-responsive nanoplatforms have been developed as an effective tumor treatment tool, greatly enhancing the tumor accumulation of the loaded therapeutic agents and facilitating the release of cargoes in the acidic tumoral microenvironment [205]. Currently, researchers typically use changes in chemical structure (such as changes in hydrophilicity through deprotonation and protonation) and acid-sensitive chemical bonds to design pH-responsive nanoplatforms (Fig. 6). Additionally, the designed pH-sensitive formulations usually have the ability to protect several therapeutic agents and vehicles for tumor therapy from being trapped in endosomes [206, 207]. Generally, the encapsulation of chemotherapeutics inside pH-responsive nanoparticles is an efficient approach for prolonging the blood circulation time of the encapsulated agents and their retention inside the nanoparticles in a physiological environment. Moreover, pH-responsive nanoparticles are also able to improve the pharmacokinetics and biodistribution of the encapsulated payload. This is essential for delaying metabolism and the subsequent release of drugs.

##### Protonation and deprotonation-based nanoplatforms

Protonation and deprotonation are widely used mechanisms for pH-sensitive nanocarriers in tumor treatment. pH-responsive nanoplatforms including polyelectrolytes, such as poly(aspartic acid-graft-imidazole), cationic poly(β-amino ester) (PBAE), anionic poly(Asp), PDMAEMA, polysulfonamide, poly(histidine) (poly(His)), and poly(acrylic acid) (PAA), are shown in Table 3. In an advanced strategy to design biocompatible nanoparticles, it has also been proposed that biodegradable materials such as enzyme-responsive chitosan and certain polypeptides can be used for protonation and deprotonation-based nanomaterials through functionalization with an acid-responsive group to the backbone of biodegradable materials [208–210]. These materials generally contain –COOH as anionic groups and –NH_2_ as cationic groups combined with other hydrophobic or hydrophilic molecules, which can be further used in pH-responsive nanoplatforms through protonation and deprotonation.

**Table 3:** pH-responsive building blocks for tumor treatment

pH-sensitive building block	Therapeutic agent	Tumor model	Therapeutic application	Refs.
Poly(2-(hexamethyleneimino)ethyl methacrylate	siBRD4-loaded TCPA2-NPs	Prostatic cancer	LNCaP-bearing mouse GT	[477]
	HRNMs	Glioblastoma	Chemotherapy	[478]
Poly(diisopropanol amino ethyl methacrylate)	GPDPA NPs	Glioblastoma	Chemotherapy PTT	[479]
Benzoic-imine bond	CA-MTX NPs	Cervical carcinoma	Chemotherapy	[480]
	nBSA-Dox	Hepatocellular carcinoma	Chemotherapy	[481]
	Nd^III^IP-N = CH-PEG	Cervical carcinoma	Chemotherapy PTT	[482]
	DOX-ICM	Glioblastoma	Chemotherapy	[483]
	Au@PP/RA/siRNA	Pancreatic cancer	Chemotherapy	[484]
Pyridine-2-imine	Gold nanomachine	Breast cancer	PTT	[485]
	PMNP-DOX@RBC	Breast cancer	Chemotherapy, CDT	[486]
Amide bond	DOX-CC-NP	Squamous cell carcinoma	Chemotherapy	[19]
	PDNBF NPs	Breast cancer	Chemotherapy PTT	[487]
Nanodrug complex	MONCs	Breast cancer	Chemotherapy PDT	[488]
	B780/Qu NPs	Breast cancer	Chemotherapy PDT, PTT	[98]
Gadolinium oxide	Gd_2_O_3_ NSs	Melanoma	Chemotherapy	[489]
	FS-GdNDs	Breast cancer	PTT	[490]
Triplex DNA sequence	NLNs/DOX	Breast cancer	Chemotherapy	[491]
	DNA Conjugated AuNPs	Breast cancer	Chemotherapy PTT	[492]

Cationic materials with -NH_2_ groups can effectively protonate in an acidic environment and show excellent hydrophilicity, while they can deprotonate in a neutral environment to show hydrophobicity. In contrast, anionic materials with –COOH groups [211–214] can also deprotonate and protonate in the opposite way. For instance, the groups of imidazole can be easily protonated under acidic conditions, as they have a pair of electrons on the unsaturated N atoms, leading to the conversion of hydrophobic to hydrophilic states, which can incur disintegration of the nanocarriers and consequently release the loaded therapeutic agents. Besides, poly(His)-PEG shows obvious nanoscale core–shell micelles in a neutral environment consisting of the hydrophilic PEG shell and hydrophobic cores of poly(His) by deprotonation. However, the protonation of poly(His) responds to His groups and destabilizes micelles because of the reduced hydrophobicity of poly(His) at pHex. Further, the poorly soluble therapeutic agents encapsulated in the core of the pH-responsive micelles can be effectively released in an acidic environment because of the destruction of the hydrophobic cores. In another example, Oh et al. also prepared pH-responsive micelles using amphiphilic polyelectrolytes for docetaxel delivery. Similarly, the prepared micelles exhibited good colloidal stability under physiological conditions, while they became unstable due to protonation of the imidazole group under acidic conditions. Therefore, the docetaxel-encapsulated micelles have pH-responsive release behavior due to structural changes induced by protonation of the imidazole groups in the amphiphilic polyelectrolytes. pH-responsive release can also lead to high stability in blood circulation, a decrease in the toxicity of healthy tissues, and increased drug availability.

Additionally, some materials with anionic polyelectrolytes have also been used to prepare pH-sensitive nanocarriers for drug delivery [215]. However, the strategy of taking advantage of anionic amphiphilic molecules to prepare pH-responsive and tumor-targeted nanocarriers can be different from that utilizing cationic materials. Under acidic conditions, such as pHen and pHex, anionic polymers containing -COOH groups can exist as protonated (hydrophobic) units and are not applicable to tumor-targeted micelles from amphiphilic polymer blocks rich in anionic groups. As a result, anionic polymers can be encapsulated with some chemotherapeutics such as DOX using hydrophobic interactions in physiological environments, and therapeutic agents can be effectively released for specific tumor treatment under acidic conditions via weakened interactions owing to protonation.

##### Acid-sensitive bond cleavage-based nanoparticles

As previously described, the acidity differences between the various compartments of tumor cells and between tumors or healthy tissues have received widespread consideration for designing pH-responsive chemical structures. In particular, acid-sensitive chemical bonds have been intensively investigated for pH-responsive nanocarriers, such as esters, imines, and hydrazine. These acid-sensitive bond-based nanoplatforms have been proven to be relatively stable in physiological environments but are easily broken via nucleophilic substitution reactions under acidic conditions [216]. Further, the acid-sensitive bonds can be directly conjugated to the therapeutic agents as labile groups in nanoparticles. For example, with the protonation of labile compounds containing C=N bonds (such as hydrazone bonds, imine groups, and oxime bonds) under acidic conditions, they can be readily susceptible to nucleophilic substitution by H_2_O because of the increased electrophilicity of carbon atoms [217–220]. Hydrazone linkages in particular, with satisfactory acid responsiveness and a fast degradation rate, have been widely used in different pH-responsive nanoplatforms such as liposomes, nanoparticles, and micelles [210, 221–225]. Additionally, acid-responsive groups can also be applied to improve the limited cargo release from the nanoparticle core and target-cell interactions because of PEGylation. For example, Wu et al. synthesized hydrazone linker-functionalized liposomes to address the problems with PEGylation. As expected, the hydrazone bond-functionalized liposomes exhibited satisfactory lysosomal escape properties and enhanced tumor accumulation in comparison to normal liposomes [226]. However, imine bonds have poor stability under physiological environments due to the loss of mesomeric effects in comparison to hydrazone bonds [227]. Therefore, researchers have made significant efforts to improve the stability of imine bonds by introducing strong *π*–*π* conjugated systems such as benzene rings [228, 229]. For example, Wang et al. prepared versatile biomimetic nanoparticles based on formyl benzoic acid-PEG-maleimide functionalized mesoporous silica against metastatic triple-negative breast cancer. After loading of immune adjuvant and photothermal therapeutic agents, the immune peptide was then linked to the surface of nanoparticles through acid-responsive benzoic-imine bonds. It was applied to the therapy of metastatic triple-negative breast cancer through immune remodeling and photothermal ablation [230]. Taken together, these low-pH-responsive nanoparticles can effectively release the encapsulated chemotherapeutic agents upon encountering the acidic TME in targeted cancer therapy.

#### Enzyme-responsive targeting strategies

Enzymes, being a significant component of the nanobiotechnology toolbox, have exceptional biorecognition abilities as well as excellent catalytic properties. Generally, abnormal enzyme expression observed in cancer provides many opportunities for designing targeted nanoparticles modified with enzyme-responsive linkages. Recently, many smart nanoparticles have been prepared for intracellular as well as extracellular tumor-specific drug delivery based upon enzyme expression at the target site.

##### MMP-responsive nanoplatforms

Matrix metalloproteinases (MMPs), which are overexpressed in various types of tumors, are closely related to cancer pathophysiology. MMP-2 and MMP-9 in particular have been explored for preparing enzyme-responsive nanoplatforms [231, 232]. For example, Yamada et al. prepared two PTX prodrugs by conjugating an octapeptide (AcGPLGIAGQ) with PTX at different sites that could be effectively broken down by MMP2 in the tumor microenvironment. These nanoparticles can effectively release PTX to inhibit cancer cell proliferation [233]. Among various natural materials, gelatin is an example of a biocompatible polymer that can be degraded by MMPs and promote cargo release in tumor sites. For instance, Wang et al. developed MMP-responsive PVA-peptide conjugates for achieving self-assembly with enhanced tumor accumulation, capable of improving PD-L1 blocking efficiency for augmented immunotherapy. Once the self-assembled nanoplatforms entered the TME, the enzyme-cleavable peptide could be immediately degraded under the action of overexpressed MMPs to effectively release cargoes for cancer treatment [234]. Furthermore, gelatin-functionalized DOX-loaded mesoporous silica nanoparticles have been applied for the delivery of therapeutic agents against MMP-9 overgenerated in cancer. As expected, the prepared nanoformulation showed clearly enhanced DOX release under simulated tumor microenvironment conditions and in tumor cell models [235]. In addition, Zhang’s group synthesized camptothecin-encapsulated mesoporous silica nanoparticles surface functionalized with targeting cRGD and MMP-2 responsive fluorescence imaging groups, acting as a diagnostic platform as well as for tumor location. These advanced nanoplatforms were able to efficiently undergo enzymatic hydrolysis in overexpressed MMP-2 environments to improve tumor treatment by the release of their cargoes [236]. Further, some amphiphilic block copolymers (such as PCL-PEG) are suitable for the preparation of versatile delivery platforms against cancer. For instance, PTX-encapsulated PEG-PCL nanoparticles functionalized with activated low molecular weight protamine showed satisfactory targeted glioma effects. Further, these advanced nanoparticles also exhibited enhanced MMP-dependent cellular internalization, increased cytotoxicity, and augmented tumor suppression in glioma models [237]. In another study, Yang et al. prepared a versatile nanoparticle based on MMP-sensitive Au nanoparticles for tumor-specific photoacoustic imaging-guided tumor treatment and drug delivery. The Au nanoparticles could be further grafted with complementary DNA strands, functionalized with PEG and conjugated with therapeutic agents through MMP-responsive peptides and thermal-sensitive linkers, respectively. As a result, the developed nanoparticles showed augmented efficiency in tumor treatment and photoacoustic imaging in comparison to MMP-inert nanoparticles [238]. Similarly, hydrophilic siRNA and poorly soluble drugs could be effectively codelivered using versatile micelles prepared by MMP-2-responsive copolymers. The prepared nanoplatform showed satisfactory colloidal stability and enhanced endocytosis efficiency in different tumor cell lines and significant passive targeting behavior in tumor-bearing models. Mallik et al. prepared an MMP-9 responsive nanoplatform using collagen-simulated lipoprotein conjugated to PEG cleavable polymers to encapsulate Gem. The designed enzyme stimuli-responsive nanoparticles demonstrated a faster Gem release rate treated with MMP-9 and a higher tumor inhibition ratio in comparison to MMP-inert nanoparticles [239]. Yang’s group [240] designed an advanced MMP stimuli-responsive nanoplatform encapsulated with the chemotherapeutic agent curcumin using a block copolymer with surface-adsorbed peptides that could improve endocytosis. The prepared nanoplatform showed a sustained curcumin release behavior under physiological conditions, while release could be accelerated under conditions that mimic the tumor microenvironment. There is no doubt that these designed MMP-responsive nanoparticles present excellent tumor specificity and therapeutic efficacy in cancer models with few side effects

##### Heparanase-responsive nanoplatforms

Glycosaminoglycans and structural proteins together constitute the matrix of the tumor tissues, of which the main component of glycosaminoglycans is heparan sulfate proteoglycan [241]. Furthermore, heparan sulfate proteoglycan is actively involved with various biological factors (including VEGF, TGF-β, and b-FGF) that play an important role in the interaction between normal cells and tumors. In addition, some reports have pointed out that highly metastatic and malignant cancers frequently over generate heparanase-1, which can degrade HSPGs in the tumor microenvironment, causing enhanced secretion of the above bioactive factors and the consecutive triggering of related pathways resulting in cancer metastasis, epithelial-mesenchymal transition, and neovascularization. Additionally, the new spaces formed in the matrix can also result in cancer invasion and metastasis [242]. It has also been reported that heparanase-1 can effectively degrade heparin, suggesting a potential novel nanovehicle with heparanase sensitivity for drug delivery.

An example of utilizing heparin-prepared nanoplatforms is to bind heparin molecules via GSH-responsive disulfide bonds to construct heparin-based nanogels [243]. Another approach involved the construction of a nanocomplex through electrostatic interactions between protamine and heparin for effective loading of positively charged therapeutic agents. Researchers have developed similar versatile nanoparticles with polyelectrolyte complexes encapsulating small therapeutic agents [244]. Isothermal titration calorimetry and real-time dynamic swelling spectroscopy have been used to explore the underlying mechanisms and principles for the fabrication of advanced nanoplatforms via intermolecular electrostatic interactions [245]. During optimization of the manufacturing process, the polyelectrolyte nanocomplex can be developed with appropriate negative surface charges and particle size [246]. 1,2-Dioleoyl-3-trimethylammonium-propane (DOTAP), a positively charged phospholipid compound widely applied to construct cationic liposomes, has been applied to encapsulate hydrophobic chemotherapeutic agents through liposomes formation [247]. When codelivered therapeutic agents nanoplatforms enter the tumor microenvironment, overexpressed heparanase-1 can rapidly recognize the outer heparin shell and cleave it, resulting in the release of cargoes for tumor cell kill [242]. This also causes the positively charged nanoparticle core to be exposed to the cancer cells, and efficient endocytosis of nanoparticles by tumor cells can be achieved with this approach.

##### Cathepsin-sensitive nanocarriers

Recently, researchers have demonstrated that a variety of cathepsins are overexpressed in different types of tumors. These are found not only in tumor cells but also in tumor-related endothelial, fibroblast, myoepithelial, and osteoclast cells as well as leukocyte cells [248]. A great number of studies have been undertaken on the design and development of cathepsin-responsive nanoplatforms, particularly on Gly-Phe-Leu-Gly, which has been commonly applied as a spacer that can be effectively degraded in overexpressed cathepsin B environments [249]. For example, Xia et al. prepared a pH and cathepsin B dual-responsive nanovaccine that specifically targeted endosomal Toll-like receptors (TLRs) for enhanced tumor vaccination. In vivo results showed excellent prophylactic and antitumor effects of the nanovaccine against tumor-bearing mice. This endosome-targeted responsive nanovaccine approach provides a promising delivery platform for adjuvants to promote the design and preparation of cancer nanovaccines [250]. Accurate assessment of cathepsin B expression in vivo may provide a potential approach for early tumor diagnosis [251]. Taking advantage of precise photoacoustic imaging, an intelligent photoacoustic probe Cypate-CBT, which could effectively assemble into cypate-containing nanoprobes in response to overgenerated GSH and cathepsin B in tumor cells, was prepared by Liang’s group [251] for the accurate and specific monitoring of cathepsin B. In comparison to unmodified Cypate, this nanoprobe showed a higher photoacoustic signal in cathepsin B-positive breast cancer models, supporting the intracellular accumulation of the nanoprobes after cathepsin B-triggered self-assembly. The cathepsin B-responsive nanoprobe can be employed as an efficient photoacoustic imaging agent for the early diagnosis and targeted therapy of cancer.

#### Hypoxia-responsive targeting strategies

Hypoxia, considered a significant hallmark of solid tumors, has been observed in more than 60% of cancers [252, 253]. The partial pressure of O_2_ (pO_2_) is generally approximately 40–60 mm Hg in normal tissues while it is less than 10 mm Hg in tumor tissues, and even as low as 0–2.5 mm Hg in some cases [254–256]. The O_2_ consumed by tumor cells exceeds supply leading to this pathological phenomenon. Abnormally vigorous metabolism and cell growth in tumor cells can deplete intracellular O_2_. Secondly, the vascular system in the tumor tissue is disordered, resulting in an insufficient supply of O_2_. Finally, the short O_2_ diffusion distance (less than 200 μm) cannot meet the demand of tumor cells further away from the blood vessels [257–259]. To better adapt to this harsh living environment, hypoxic cancer cells must alter some of their biological characteristics, such as upregulating the levels of HIF-1α, carbonic anhydrase IX (CA IX), and other enzymes [260].

As a result, enhanced cancer metastasis and poor therapeutic effects are usually evident in hypoxic-stimulated tumors [261, 262]. For instance, the hypoxic microenvironment can result in overexpression of HIF-1α, which is capable of modulating gene expression relevant to tumor proliferation, invasion and metastasis to facilitate the resistance to O_2_-dependent antitumor strategies, such as chemotherapy, PDT and RT [263–266]. In addition, hypoxia-adapted tumor cells generally do not have the rapid division characteristics of normal tumor cells, so they are not sensitive to conventional chemotherapeutic agents that interfere with DNA replication [267, 268]. Further, O_2_ plays an important part in repairing DNA dysfunction after radiation treatment (such as X-ray and γ-ray) during RT, and it is the source of PDT or SDT-mediated ROS applied to fight tumors [267, 269–273]

Hypoxia of tumor tissue is generally considered to indicate poor prognosis for tumor treatment, but particular biological features can make it a specific target for cancer therapy. In fact, tumor cells prefer aerobic glycolysis to obtain energy rather than the conventional oxidative phosphorylation pathway due to the Warburg effect. Therefore, many enzymes related to electron donation or reduction response are overgenerated in hypoxic tumor cells, such as azoreductase, nitroreductase, methionine synthase reductase, inducible nitric synthase and DT-diaphorase (DTD) [274, 275]. Hence, considerable efforts have been made to develop hypoxia-responsive nanoplatforms that can be activated by these enzymes for enhanced tumor treatment based on the above findings [274, 276, 277]. Hypoxia-responsive chemical bonds (Table 4), including nitro groups, azo groups, quinone and N-oxide compounds, are also applied in the construction of hypoxia-responsive nanoparticles. They can change their conformation and physicochemical characteristics such as hydrophobic features and electron affinity by gaining or losing their electrons [278, 279]. As anticipated, such hypoxia-responsive nanoparticles have been found to exhibit satisfactory performance for drug delivery. They have great potential for tumor treatment including hypoxia-responsive cargo release, prolonged blood circulation time, and enhanced tumor penetration and accumulation. Below we discuss the chemical structures that can be used to design effective hypoxia-responsive nanoparticles and the strategies for taking advantage of these nanoparticles for enhanced tumor treatment.

**Table 4:** Hypoxia-selective chemical bond-triggered nanoplatforms

Type of chemical bond	Therapeutic agent	Therapy method	Tumor model	Refs.
Nitro	DOX/CP-NI NPs	Chemotherapy, PDT	Cervical carcinoma	[505]
	DOX@HMs	Chemotherapy, RT	Breast cancer	[506]
	DOX/FOBD liposome	Chemotherapy	Cervical cancer	[507]
	HRNP/siRNA	Chemotherapy	Breast cancer	[286]
	HC/PN/DOX NPs	Chemotherapy, PDT	Lung cancer	[508]
	NCs/DOX + Ce6 micelles	Chemotherapy, PDT	Breast cancer	[509]
	ALP-(MIs)n/DOX	Chemotherapy, RT	Glioma	[510]
	Gd-Au DENPs-Nit	RT	Nasopharyngeal carcinoma	[511]
Azo	DOX@AMOFs@ DRHC/CPPs	Chemotherapy	Breast cancer	[306]
	mPEG-AzoPAsp-IM micelles	PDT	Lewis lung carcinoma	[294]
	DOX@NP	Chemotherapy	Lung cancer	[512]
	CPs-CPT-Ce6 NPs	Chemotherapy, PDT	Cervical carcinoma	[300]
	PEG-Azo-PEI-DOPE	Chemotherapy	Cervical carcinoma	[513]
	CAGE	Immunotherapy, PDT	Melanoma	[514]
	ALN-HR-PMs/DOX	Chemotherapy	Prostate cancer	[320]
N-oxide	TPZ/UCSs	Photodynamic/Chemo/ immunotherapy	Colorectal cancer	[515]
	HAS-GOx-Fe^3+^-TA (HGTFT)	Chemotherapy, CDT	Breast cancer	[516]
	TENAB NPs	Chemotherapy, PTT, PDT	Cervical carcinoma	[517]
	Lip/Ce6/TPZ NPs	Chemotherapy, PDT	Breast cancer	[518]
	UiO-66-H-P NMOFs	Chemotherapy, PDT	Glioblastoma	[519]
	HA@AQ4N-Cu (II)-gossypol NPs	Chemotherapy	Prostatic cancer	[520]
	YS-DMONs-AQ4N- GOx	Chemotherapy	Prostatic cancer	[326]
	Mn-APPMSF	Chemotherapy, PTT	Hepatocellular carcinoma	[521]
	AQ4N-^64^Cu-hCe6- liposome	Chemotherapy, PDT	Breast cancer	[325]

##### Hypoxia-responsive drug delivery

###### Nitro compounds

In hypoxic cancer cells, the -NO_2_ group can be effectively converted into -NH_2_ via a series of biochemical reactions involving NADPH and nitroreductase. It should be noted that the first intermediate -NO can be reversely oxidized to the original -NO_2_ under normal conditions [277]. Based on the above bioreduction reactions, polymers with -NO_2_ groups (such as 2-nitroimidazole and nitrobenzyl alcohol) have been applied to design hypoxic-responsive nanovehicles for the delivery of therapeutic agents. One significant profile of 2-nitroimidazole is the conversion from a hydrophobic to hydrophilic state after its reduction to 2-aminoimidazole in a hypoxic environment. If functionalized with a hydrophilic block copolymer, the hydrophobic nitroimidazole groups can allow the block copolymer to form encapsulated therapeutic agent nanocarriers through intermolecular hydrophobic interactions. However, the hydrophobic nitroimidazole groups can be effectively transformed into hydrophilic aminoimidazole groups, leading to disassembly of nanocarriers and the release of loaded therapeutic agents in hypoxic tumor cells. For example, Thambi et al. developed hypoxic-responsive nanoplatforms based on nitroimidazole-functionalized block co-polymers for encapsulating and controlling the release of therapeutic agents [280]. As expected, the cumulative release of therapeutic agents from the designed nanoplatforms was relatively slow under normoxic conditions but was obviously accelerated under hypoxic conditions. In addition to the transformation from a hydrophobic to a hydrophilic. Currently, researchers have also developed other strategies to take advantage of the hypoxia-sensitive potential of nitroimidazole [281–283]. Tseng et al. [284] reported bioreduction-responsive nanoplatforms functionalized with HA conjugated with 6-(2-nitroimidazole)hexylamine to encapsulate lactate oxidase and a virus for use in tumor therapy. In this nanoparticle, lactate oxidase can oxidize lactate resulting in O_2_ depletion inside tumor cells. Subsequently, bioreduction of the 2-nitroimidazole of the nanocarriers converts it into a hydrophilic group and dissociates the carrier backbones to release the anticancer virus. Furthermore, Shi et al. [285] also designed a nanocarrier by co-assembly of 2-nitroimidazole-functionalized peptides and cationic lipid-like copolymers for siRNA delivery to silence the expression of a hypoxia-relevant protumorigenic gene (CDC20) against breast cancer.

###### Azobenzene (AZO) compounds

In hypoxic environments, AZO compounds can be effectively reduced by NAD(P)H quinone dehydrogenase 1 (NQO1) and azoreductase into two separate aniline groups, rendering them suitable for preparing hypoxic-responsive nanoplatforms [286–288]. Moreover, matching the hypoxia-responsive features and the broad absorption wavelength of the AZO groups with the therapeutic bio-optical window can lead to more efficient stimulus responses. Therefore, the AZO group has been applied as an ideal linker allowing biological rupture under appropriate hypoxic stimulus conditions [289–292]. For these hypoxic stimuli-responsive nanoplatforms, the AZO groups are generally used to link hydrophilic and hydrophobic moieties in amphiphilic molecules, which can self-assemble into nanoparticles under physiological conditions and disassemble to release the loaded contents under hypoxic conditions by breaking the AZO groups [293–297]. Therefore, breakage of the AZO linker can cause the cleavage of hydrophilic groups when the designed nanoparticles reach the hypoxic tumor microenvironment, leading to enhanced cellular internalization and tumor accumulation of nanoparticles [294, 298, 299]. For example, Zhang et al. [300] synthesized a hypoxic-degradable nanocarrier functionalized with AZO-containing hydrophobic groups to encapsulate the chemotherapeutic agent camptothecin and photosensitive therapeutic agent chlorin e6 for laser-augmented synergistic chemo-photodynamic therapy. In this designed nanoplatform, chlorin e6-mediated PDT can exacerbate tumor hypoxia, allowing the hypoxia-responsive nanocarriers to rapidly disintegrate and release the encapsulated camptothecin. Continuous O_2_ consumption during PDT or SDT can mediate an extremely hypoxic environment, giving potential for the design of azoreductase-triggered nanoplatforms acting in the local tumor region [301–304]. For example, Zhang et al. [300] reported a versatile AZO-based nanoplatform resulting in a synergistic action of chemo-photodynamic therapy. Because of the O_2_ consumption stimulated by PDT, Azo groups in the nanoparticles can be effectively cleaved by overexpressed azoreductase to trigger a faster release of chemotherapeutics in hypoxic microenvironments. Using a similar strategy, Huang et al. [305] developed smart supramolecular micelles to codeliver a photosensitizer and hypoxia-sensitive prodrug to enhance the antitumor effects. Satisfactory cancer cell killing in vitro and vivo demonstrated that the designed micelles not only offered a new platform for the codelivery of therapeutic agents to tumors, but also provided novel ideas for designing and preparing advanced materials for multimodality tumor treatment.

Azoreductase-sensitive organic ligands can also be used to construct nanoscale coordination complexes and bring hypoxia-sensitive characteristics to nanotheranostics for tumor diagnosis and treatment. For example, Huang et al. [306] prepared an azoreductase-triggered nanocomplex where the encapsulated chemotherapeutic DOX and siRNA were capable of downregulating the expression of HIF-1α, thus reducing multidrug resistance. 4,4′-Azobisbenzoinc acid, as the main ligand of the nanocomposites, was effectively reduced by azoreductase to release the encapsulated DOX and siRNA in the hypoxic tumor cells. In another example, Zhou et al. [292] reported on an aptamer/antibody nanoformulation functionalized with hypoxia-sensitive AZO compounds capable of decreasing off-target effects. In this nanoformulation, a conditional aptamer was conjugated with hydrophilic polymers containing AZO groups which played an important role in preventing binding to normal cells. The hydrophilic block polymers could be detached from the nanoparticles through the reduction of AZO, allowing aptamer/antibody recognition of the cancer cell surface in a hypoxic microenvironment. Mesoporous silica nanoparticles are another important platform as inorganic drug delivery carriers for tumor treatment. They have the advantages of low side effects, good biocompatibility and stability, relatively uniform size and a large specific surface area [307, 308]. For example, Jang and colleagues developed hybrid mesoporous silica nanoparticles functionalized with β-cyclodextrin and 4-(phenylazo) benzoic acid for improved on-demand drug release. As expected, the nanoparticles displayed improved selective drug release and significant cytotoxicity in comparison to nonresponsive nanoparticles [309].

###### Oxide groups

The N-oxide group can also be used in the design and preparation of hypoxia-responsive nanoplatforms for effective tumor treatment [310]. Tirapazamine and banoxantrone dihydrochloride are the most studied agents. Tirapazamine is an aromatic N-oxide compound while banoxantrone dihydrochloride is an aliphatic N-oxide derivative, exhibiting higher cytotoxicity in hypoxic cancer cells than in normal cells [311–315].

In hypoxic tumor cells, tirapazamine can produce radical species that break DNA through a single-electron reduction reaction catalyzed by various intracellular reductases, leading to irreversible damage and apoptosis [316]. Because of the specific responsive strategy, nanoparticles that elevate tumor hypoxia can significantly enhance the antitumor effects of tirapazamine [317]. Guo et al. designed a novel nanoplatform capable of regulating the tumor microenvironment via Fenton reaction-based chemodynamic therapy. This nanoplatform utilizes glucose-mediated continual O_2_ consumption to create a localized hypoxic microenvironment for enhanced tirapazamine-mediated chemotherapy. The production of exogenous H_2_O_2_ by GOx facilitates the release of Fe^3+^ from the nanoparticles to convert H_2_O_2_ into the highly cytotoxic ∙OH. This versatile nanoplatform showed enhanced tumor accumulation and excellent antitumor efficacy in tumor-bearing models [318]. Yang et al. prepared nanoparticles capable of enhancing tumor hypoxic levels by loading vascular disruption agents that cut off the O_2_ supply. As a result, the designed nanoparticles not only suppressed tumor proliferation but also effectively inhibited tumor metastasis [319]. Moreover, PDT can be used as an excellent strategy to enhance tumor hypoxia and improve tirapazamine-mediated chemotherapeutic effects through the transformation of ^3^O_2_ to ^1^O_2_. For example, Yan et al. encapsulated tirapazamine into the pores of porphyrinic-based MOFs on the surface of lanthanide-doped upconversion nanoparticles to prepare a versatile nanotheranostic agent. The cell and animal experiment data showed that the combination of tirapazamine and PDT yielded enhanced therapeutic efficacy. Further, the integration of nanotheranostic agents with anti-programmed death-ligand 1 (anti-PD-L1) clearly decreased the tumor volume at distant sites by improving immune infiltration [320].

Banoxantrone dihydrochloride (AQ4N) can not only be selectively activated in hypoxic tumor cells but can also be reduced under the action of reductases [321, 322]. The protonated form (1,4-bis([2-(dimethylamino-N-oxide)ethyl]amino)5,8-dihydroxy-anthracene-9,10-dine (AQ4) containing two tertiary amine groups) can utilize DNA intercalation to strongly suppress topoisomerase II [323, 324]. Thus, Feng et al. prepared a multipurpose liposome to encapsulate soluble banoxantrone dihydrochloride and poorly soluble ^64^Cu-hCe6 into the cavity and lipid layer of the liposomes, respectively. Severe local hypoxia induced by Ce6 under laser irradiation could activate the anticancer activity of banoxantrone dihydrochloride, resulting in improved therapeutic efficacy in tumor-bearing models [325].

In another study, Yang et al. prepared novel organosilica nanoparticles containing tetrasulfide bonds to encapsulate banoxantrone dihydrochloride and GOx for tumor treatment [326]. Overexpressed GSH in tumor cells can effectively cleave the tetrasulfide bonds to disrupt the nanoparticles leading to the release of banoxantrone dihydrochloride and GOx. Subsequently, GOx can consume O_2_ and glucose to produce H_2_O_2,_ thereby exacerbating the hypoxia and further promoting the transformation of banoxantrone dihydrochloride into highly toxic AQ4. Furthermore, the consumption of GSH through the action of tetrasulfide bonds can greatly enhance oxidative stress leading to tumor cell death. This combinatorial strategy showed satisfactory in vivo and in vitro results.

The near-infrared fluorescence of banoxantrone dihydrochloride also plays an important role in monitoring therapeutic agent release and biodistribution for tumor diagnosis and treatment. Shen et al. [327] prepared multifunctional carrier-free nanoparticles loaded with banoxantrone dihydrochloride to realize fluorescence imaging-guided tumor treatment. There was no obvious fluorescence when banoxantrone dihydrochloride was encapsulated into nanoparticles due to aggregation-induced quenching. However, strong fluorescence was observed after the collapse of the nanoparticles which released banoxantrone dihydrochloride into the acidic tumor microenvironment.

###### Quinone compounds

Quinone compounds and their derivatives have been used in developing responsive tumor treatments because of their excellent electronic and chemical characteristics, particularly in hypoxia-activated prodrugs and fluorescence imaging probes. Due to the particular redox potential properties of quinone compounds, they can produce semiquinones or hydroquinones via one or two-electron reduction, respectively [328]. For example, the elimination of indolequinones can be achieved under hypoxic environments with the aid of the DT-diaphorase NQO1, which is overexpressed in various cancer cells and plays a crucial role in bioreduction [329, 330]. Taking advantage of this property, Tanabe et al. [331] designed ^19^F nuclear magnetic resonance (NMR) monitor nanoprobes to detect the biological reduction effects of indolequinones. A single new signal was observed when the nanoprobes were incubated with β-NADPH and NADPH-dependent cytochrome P450 reductase in hypoxic environments in comparison to the preincubation groups. This hypoxic-responsive probe could become a valuable candidate for magnetic resonance imaging of cancers. In addition, Jiho et al. reported on an enzyme-responsive prodrug generated by the chemical bonding of dopaquinone and 5-fluorodeoxyuridine. The results of in vitro assays demonstrated that this prodrug increased the hypoxia targeting capability of 5-fluorodeoxyuridine while significantly reducing the cytotoxicity to normal cells [332]. Cho et al. developed versatile nanocarriers functionalized with benzoquinone groups [333] for the redox-responsive release of therapeutic agents. Overall, there is clearly potential for using the unique characteristics of these compounds and their derivatives for the design and development of novel hypoxia-sensitive nanoplatforms for enhanced tumor treatment.

##### Hypoxia-responsive O_2_ release

The effective delivery of O_2_ to the tumor microenvironment shows great potential in tumor therapy. Hyperbaric oxygen has been applied to enhance the O_2_ concentration and reduce the side effects of hypoxia during radiotherapy [334, 335]. However, some adverse effects of hyperbaric oxygen, including hyperoxic seizures and barotrauma, have limited its clinical application [336–339]. Nanoparticles offer alternatives for the precise delivery of O_2_ to the TME where it can be effectively released and diffused into hypoxic lesions. Perfluorocarbon has been widely applied for the construction of versatile nanoparticles that can carry O_2_ which it can dissolve. Song et al. [340] reported the surface modification of nanoparticles with the radiosensitizer tantalum oxide (TaOx) and functionalization of the nanoplatforms with PEG. These designed nanoparticles highly enhanced tumor cell oxygenation and solved the problems of RT in in vivo models. Hemoglobin, rich in red blood cells (RBCs), has been commonly applied as an O_2_ carrier because of its excellent O_2_-carrying capability [341–343]. For instance, Liu et al. [342] prepared versatile nanoparticles engineered from recombined RBC membranes for the integration of hemoglobin and other therapeutic agents to enhance therapeutic efficacy. The extreme hypoxic microenvironment in tumor cells can effectively promote the release of O_2_ from the designed nanoparticles. As expected, these nanoparticles significantly alleviated the hypoxic environment and enhanced the therapeutic efficacy. Overall, such chemical approaches can be used to design effective hypoxia-targeted nanoparticles for enhanced tumor treatment.

##### Hypoxia-mediated O_2_ production

Enhancing O_2_ production in the hypoxic tumor microenvironment has been considered another important approach to address problems with radio- and photodynamic therapy. Utilizing the significant characteristics of the hypoxic tumor microenvironment, including high redox potential and acidic conditions, nanoplatforms can effectively produce O_2_ in situ by the catalysis of H_2_O_2_. This approach can be divided into two categories: the utilization of the high intracellular H_2_O_2_ levels in the hypoxic tumor microenvironment and the utilization of carrying groups to generate H_2_O_2_ locally. Metal nanoparticles are commonly applied in catalysis [344, 345], imaging [346–348], and medical applications [349, 350] because of their high specific surface area, nanoscale size and unique physicochemical profiles. Under the acidic conditions of the hypoxic tumor microenvironment, the catalytic ability of many metal nanoparticles can be activated to transform H_2_O_2_ into O_2_ and H_2_O or hydroxyl radicals. Various theranostic nanoagents have been developed based on manganese (Mn) to induce Fenton-like reactions.

Recently, Chen et al. designed an excellent TiO-porphyrin-based nanoplatform (FA-TiOPs) by loading TiO-porphyrin in FA-modified liposomes. These liposomes could effectively catalyze H_2_O and overexpress H_2_O_2_, in situ producing active ROS. Furthermore, TiO-porphyrin could photo-split H_2_O to generate H_2_O_2_, ∙OH radicals, and O_2_. The increased O_2_ concentration not only alleviated the hypoxic tumor microenvironment but could also be further converted by TiO-porphyrin into ^1^O_2_ to kill cancer cells. Furthermore, researchers have reported that the high energy of TiO-porphyrin in the excited state and the narrow gap energy between the triplet excited state and the excited state may facilitate effective photocatalytic reactions. In addition, overgenerated H_2_O_2_ in tumor cells could also be catalyzed to produce ^1^O_2_, particularly in an acidic environment, exerting active antitumor effects and preventing damage to healthy tissues. Overall, tumor-targeted liposomes provided adequate ROS to the tumor through several in situ photocatalytic reactions that are O_2_ dependent and achieve effective cancer inhibition. Other therapeutic agents such as immunostimulatory or chemotherapy drugs can also be encapsulated into such hypoxia-responsive nanoparticles through conjugation or loading strategies for effective tumor-targeted therapy.

##### Other hypoxia-responsive nanoplatforms

With increasing attention being paid to the hypoxic TME, a growing number of strategies have been developed using external stimuli, such as lasers. Xu et al. [351] designed an NIR laser-controlled O_2_/Pt^2+^ self-producing prodrug (UCPP) to enhance PDT efficacy in the hypoxic TME allowing combined photo-chemotherapy. The nanosystem included Pt^4+^ and Ce6, in which upconversion nanoparticles were encapsulated to transform 980 nm near-infrared light into 365 nm and 660 nm emissions to decompose Pt^4+^ and initiate Ce6-mediated PDT. The decomposition of Pt^4+^ produced O_2_ for depletion in the PDT process and released Pt^2+^ for chemotherapy. Therefore, this novel nanosystem achieved enhanced tumor accumulation and satisfactory tumor suppression in mouse xenograft models with no recurrence.

Another emerging method is the use of lasers to decompose abundant water molecules in living organisms to relieve hypoxic TME. Thus, Zheng et al. [352] prepared C_3_N_4_-based versatile nanoparticles to trigger the decomposition of H_2_O and produce O_2_ after exposure to 630 nm laser irradiation to reverse hypoxia-mediated PDT tolerance. In another example, Jiang et al. [353] reported that prepared ultrathin graphdiyne oxide nanoparticles could also decompose water to generate O_2_ following laser irradiation, and subsequently transform O_2_ into toxic ^1^O_2_ for tumor kill. The excellent photothermal conversion efficiency significantly enhanced blood circulation to the hypoxic tumor microenvironment. Additionally, Tang et al. designed a nanosensor for the tracking and assessment of non-small cell lung cancer using near-infrared excited hypoxia imaging in which the acceptor and donor pairs within a biological MOF matrix are precisely controlled to rationalize upconversion Förster resonance energy transfer. It was found to be beneficial both in vitro and in in vivo zebrafish models.

To overcome the limitations of hypoxia treatment using photodynamic therapy, several radical generators [354] that do not consume O_2_ have been developed. For example, Dong et al. [355–357] have developed several advanced organic superoxide radical photo-generators to effectively address problems existing in hypoxia treatment with PDT. In one instance, they formed highly efficient photosensitizers to carry out type I PDT elimination of hypoxic tumor tissues by vascular disruption. The in vitro and in vivo results showed that these nanoparticles could not only overcome the hypoxia paradox but also suppress cancer metastasis through treatment with type I PDT in 4TI breast cancer cell mouse models [355]. pH-sensitive zinc (II) metalated porphyrin nanoparticles were also prepared by this group to track and treat cervical cancer tumor-bearing mice. Interestingly, they observed that the phototherapy effects of the prepared nanoparticles could be effectively activated by increased acidity [357]. Taken together, these hypoxia-responsive nanoparticles are making substantial progress in targeting tumor sites and enhancing therapeutic efficacy.

#### Interstitial fluid pressure (IFP)-related targeting strategies

The IFP in healthy tissues is only about 0–3 mm Hg, while tumors show an IFP of around 5–130 mm Hg [358]. It should be noted that interstitial fibrosis and abnormal lymph vessels and blood are considered the primary reasons for increased IFP [358, 359]. Elevated IFP can serve as an obstacle to the delivery of therapeutic agents, because of drops in convection between the extravascular and intravascular spaces, resulting in restricted drug delivery to the tumor tissues. Further, it also correlates with high recurrence rates in some tumors (such as gynecological cancers) [360, 361]. Recently, some preliminary studies have reported that appropriate hyperthermia can effectively decrease intratumoral IFP to facilitate tumor treatment. The intravenous administration of chemotherapeutic agent-encapsulated liposomes has been applied in combination with two ablative heating approaches to appropriate hyperthermia and coagulative ablation. For example, Zhao et al. reported that a two-step ablation (45 ℃ for 2 min and 70 ℃ for 3 min) in conjunction with liposomes could obtain a survival benefit in comparison to administering nanoformulations with a single heating approach in a Balb/c mice bearing 4T1 tumor model [359]. Designing a versatile hyperthermia therapeutic nanoparticle appears to provide a promising potential approach for the improvement of the targeted drug delivery.

#### ATP-responsive targeting strategies

ATP, which has been called “the energy currency of the cell,” is fundamental to various cellular signal cascades. ATP concentrations can reach up to 10 mM in tumor cells, while it is only approximately 5 mM in the extracellular fluid. Therefore, a concentration gradient of ATP levels between extracellular and intracellular levels has been used to develop ATP-responsive nanoplatforms for tumor treatment. For instance, Kataoka’s group reported ATP-responsive micelles for the delivery of siRNA to tumors. Because of competitive binding between micelles and ATP, the designed micelles could be crosslinked with extracellular ATP but collapsed because of intracellular ATP, resulting in the efficient release of loaded siRNA [362]. Aida et al. developed protein-based nanoplatforms to release ATP-sensitive agents for tumor treatment. The nanocarrier was prepared using various barrel-shaped chaperonin groups assembled via coordination with Mg^2+^ into tubular structures that protected loaded therapeutic agents from biological metabolism and degradation [363]. Upon internalization by tumor cells, hydrolysis of ATP to form ADP can trigger protein conformational changes and collapse of the nanoparticles, resulting in the selective release of the contents [363].

ATP ligands have been developed for monitoring ATP using several sensors, including electrochemical, colorimetric, and fluorescent platforms [364–368]. Wang et al. prepared nanoparticles complexed with PEI hybridized with the ATP-responsive ligands, siRNA and DOX. The prepared nanoparticles using a gradient of ATP concentrations showed rapid cargo release in an ATP-responsive manner. An enhanced anti-proliferative effect was observed, possibly due to enhanced cell apoptosis in mitochondria-mediated pathways and cell cycle arrest at the G2 phase [369]. In another example, Gu et al. designed ATP-binding aptamer DNA functionalized with polymeric nanocarriers encapsulated with chemotherapeutics for targeted delivery to ATP overgenerated environments. In comparison to non-ATP responsive nanogels, the ATP-responsive nanogels achieved significant therapeutic effects in various tumor cell lines. Furthermore, functionalization with hyaluronic acid for tumor-specific targeting accompanied by ATP responsiveness improved tumor inhibition in tumor-bearing mouse models [370]. Tang and co-workers prepared switchable aptamer micelle flares conjugated to a diacyl lipid chimera, which can monitor intracellular ATP [371]. These micelles showed benefits for cell permeability and molecular imaging, with potential for tumor diagnosis and targeted delivery. In summary, ATP can be considered an efficient stimulus to promote release of preloaded drugs from nanoparticles for specific cancer treatments and for diagnostic purposes

### Exogenous stimuli-responsive targeting strategies

#### Temperature stimuli-responsive targeting strategies

Temperature stimuli-responsive nanoplatforms have been designed for tumor treatment. Ideal temperature-responsive materials with a lower critical solution temperature include poly(2-oxazo line)s (POxs), poly-*N*-isopropylacrylamide (PNIPAAm), poly(methyl vinyl ether) (PMVE), and poly(vinyl caprolactam) (PNVCL), which can readily undergo solid-to-liquid phase transitions according to external conditions [372–376]. Among these materials, PNIPAAm has been most commonly used for preparing nanoplatforms as it has a lower critical solution temperature of approximately 30 ℃. As an example, Grüll and co-workers prepared temperature stimuli-responsive liposomes loaded with therapeutic agents for high intensity focused ultrasound-mediated targeted delivery. As expected, temperature stimuli-responsive release of cargoes was observed along with enhanced endocytosis of therapeutic agents by the tumor cells [377]. In another example, Deng et al. prepared DOX-encapsulated temperature stimuli-responsive liposomes surface functionalized with iRGD peptide (CCRGDKGPDC) for targeted tumor treatment. In combination with high intensity focused ultrasound-mediated temperature stimuli-responsive DOX release, the designed liposomes were specifically internalized by αvβ3-positive tumor cells, with good treatment efficacy [378]. As explained above, temperature stimuli-responsive nanoparticles can have a significant impact at the cellular level, which potentiates the cytotoxicity of certain active pharmaceutical ingredients, mostly explained by changes in the pharmacokinetics of the agent under hyperthermic conditions and associated cellular changes, resulting in increased nanoparticle uptake.

#### Magnetic stimuli-responsive targeting strategies

Over the past few decades, magnetic stim [379–381]. Moreover, considering that magnetic nanoparticles have excellent physiochemical performances and biological effects, they have been proposed as ideal platforms for a number of tumor theranostics [382]. Encapsulated superparamagnetic Fe_2_O_3_ nanoparticles (SPIONs) present an excellent magnetic moment and satisfactory biocompatibility in comparison to other magnetic stimuli-responsive nanoplatforms. Furthermore, magnetic molybdenum disulfide (mMoS_2_) can be modified by liposomes with a phospholipid bilayer membrane structure to construct magnetically responsive nanoplatforms which do not easily aggregate in physiological solutions, and have good biocompatibility, thereby showing great promise for nanomedicine applications [383].

Successful application of magnetically responsive nanoplatforms includes encapsulation/immobilization of therapeutic agents into magnetic-responsive nanoplatforms, injection of the magnetic stimuli-responsive nanoplatforms into the body and taking advantage of external magnetic fields to recruit and activate the magnetic stimuli-responsive nanoplatforms at the lesions of interest [384–386]. Recently, Shuai et al. [387] reported a GSH-responsive MOF to effectively load IDO inhibitor, and NO donor s-nitrosothiol groups for improving antitumor immunotherapy. In this nanoplatform, the high T1 relaxivity endows magnetic resonance (MR) imaging capabilities to detect the in vivo biodistribution of nanoagents. Shi et al. reported a versatile nanodiagnostic based on DOX-encapsulated tannic acid-Fe networks (TAFs) functionalized with fibronectin for combination cancer treatment under the guidance of MR imaging. In this system, the TAF network allows the nanodiagnostic to have excellent r_1_ relaxivity for T1-weighted MR cancer imaging [388]. The development of magnetic stimuli-responsive nanoparticles with imaging properties will help to determine when there is good tumor accumulation.

#### Ultrasound stimuli-responsive targeting strategies

Ultrasound has become an excellent external stimulus capable of facilitating the disruption of nanoparticles and releasing their cargoes at the lesions of interest [389]. Ultrasound stimuli-responsive nanoplatforms can therefore be a valuable tool for enhancing therapeutic agent accumulation in tumors with low EPR effects. For example, SDT-based nanoparticles capable of continuous production of CO_2_ have been recently developed to accomplish ultrasound-mediated inertial cavitation (UIC) to augment ROS accumulation against cancer [390]. The in vitro and vivo results indicated that continuous UIC accelerated a massive generation of ROS, resulting in the improvement of SDT using a single nanoplatform. Furthermore, the highly-accumulative ROS arising from continuous UIC have been shown to induce robust immunogenic cell death (ICD), which is typically represented by increased antigen exposure and presentation, enhanced DC maturation and more activated CD8^+^T cell infiltration in tumors [391]. Price et al. prepared cisplatin encapsulated solid lipid nanoparticles guided by ultrasound stimuli. Under ultrasound treatment, the designed nanoparticles exhibited satisfactory drug release, cellular uptake and other pharmacokinetic characteristics, as well as superior antitumor efficacy in glioma models [392]. Zheng’s group prepared ultrasound stimuli-responsive DOX-loaded mesoporous silica nanoparticles featuring the ultrasound-responsive release of cargoes for glioma treatment. They showed an obvious suppression in tumor invasiveness and growth, as well as increased survival in a mouse glioma model [393]. Muragaki’s group developed epirubicin-encapsulated micelles as tumor sonosensitizers. Using HIFU, the nanoparticles could be disrupted to allow drug release in canine spontaneous chondrosarcoma, osteosarcoma, hepatocellular and prostate cancer [394]. Biocompatible piezoelectric nanoparticles have also been encapsulated with DSPE-PEG and modified with anti-HER2 Ab for targeted breast cancer treatment. As anticipated, these designed ultrasound stimuli-responsive nanoplatforms can effectively release encapsulated active pharmaceutical ingredients in a controlled manner, interfering with cell division and inhibiting tumor proliferation

#### Laser stimuli-responsive targeting strategies

Laser stimuli can break light-sensitive functional bonds or groups, including coumarinyl ester, truxylic acid and pyrenyl methyl ester. Much work has focused on employing these laser-responsive nanoplatforms to deliver chemotherapeutic agents by destroying the nanovehicle at the lesions [395–399]. For example, Chen et al. prepared a photolabile spherical nucleic acid for light-responsive codelivery of antisense oligonucleotide and siRNA. Upon exposure to an NIR laser, the prepared nanoplatforms rapidly oxidized and dissociated with continuous responsive release, resulting in a positive effect on tumor treatment [400]. Xu et al. designed a light stimuli-responsive nanoparticle to achieve long blood circulation, enhanced tumor accumulation and penetration, and rapid body elimination in an imaging-guided treatment. After the nanoplatform accumulated in the tumor regions, the cargoes could be effectively released by laser irradiation. Importantly, the released therapeutic agents could effectively penetrate the whole tumor tissue with a diameter of approximately nine millimeters giving tumor suppression [401]. Additionally, Kim’s group prepared a laser-responsive and biomimetic nanoplatform for deep tumor penetration [402]. In this study, the in vitro drug release profile and tumor cell inhibition rate were significantly improved after laser irradiation. The use of lasers as an exogenous stimulus can effectively improve the therapeutic effect and reduce the side effects by controlling the drug release behavior.

## Hybridization and combination of cancer nanomedicine

Designing smart targeting nanoparticles with stimuli-responsive profiles has proved promising for providing site-specific, accurate, and systemic drug administration. Moreover, stimuli-responsiveness can substantially increase the diverse utility of such systems by integrating drug administration with other features, such as sensing, imaging, or monitoring. The designed smart nanoparticles can accumulate in the tumor region through either passive targeting behavior (EPR effects) or receptor-mediated active targeting strategies. Subsequently, such nanoparticles provide yet another possibility to fine tune their response toward each stimulus individually, enabling drug release to be precisely controlled under the cumulative effect of multiple stimuli. In these nanoparticles, multiple impulses are integrated to activate nanoparticles in the TME by introducing exogenous stimuli, such as laser and ultrasound. In such systems, one of these stimuli will be employed to load the drug into the nanoparticles and trigger the drug release. Additionally, the activation of drug release under external stimuli, including a magnetic field, temperature, light or ultrasound, can also be achieved at the targeted site. Owing to the complex TME, including the abnormal expression of multiple receptors, high redox potential, and abnormal metabolic conditions, smart nanoparticles have been specially developed for anticancer medication. Studies using stimuli-responsiveness and targeting strategies are detailed in Table 5.

**Table 5:** The hybridization and combination of cancer nanomedicine

Targeting strategy	Stimuli-responsiveness	Therapeutic agent	Tumor type	Refs
FA	pH	PEG-FA/(DOX + VER)@ZIF-8	Melanoma	[522]
	GSH	FA-S–S-PLGA NPs	Lung cancer	[523]
	ROS	Lut/FA-Oxi-αCD NPs	Breast cancer	[524]
	MMP2	F/TMSP-NLC	Fibrosarcoma	[525]
	pH and GSH	PsEEL-DOX/PTX NMs	Lung cancer	[403]
	pH and ROS	DT-NP	Breast cancer	[527]
	pH and laser	HM-Bi@PEG-FA NSs	Lung cancer	[528]
HA	GSH	HL/MOS@M780&LOD NPs	Breast cancer	[526]
	pH	HA/(R837 + 1 MT)@ZIF-8	Melanoma	[529]
	Laser	DOX/ICG-CuS@MnO2/HA NPs	Breast cancer	[530]
	pH and GSH	DOX/siGCN5@HPMSNs	Breast cancer	[531]
	GSH and hypoxia	PaHAsC	Melanoma	[91]
RGD	GSH	RGD/MoS_2_/DOX	Cervical cancer	[532]
	pH	Met/GOx@His/ZIF-8∼RGD	Breast cancer	[533]
	MMP-2	RHMH18@AuD NPs	Ovarian cancer	[534]
	Laser	SPIOCs@HSA(PTX)-RGD	Glioma	[535]
	pH and esterase	IR825@IRI-ATRA/RGD NPs	Breast cancer	[536]
	pH and GSH	CuS DENPs	Breast cancer	[537]
Biotin	pH	B780/Qu NPs	Breast cancer	[98]
	GSH	SS-biotin-Ppy NWs	Breast cancer	[538]
Transferrin	Temperature	TMNP	Breast cancer	[80]
	GSH	DMSN@PMAsh-Tf	Lung cancer	[539]
	pH/temperature	LF-PNIPAM-co-AA	Breast cancer	[540]
LHRH	GSH	PTX-LHRH-DCMs	Breast cancer	[97]
	pH, HIFU, and ultrasound	LHRH-ELP-DOX	Breast cancer	[541]
EPR effects	pH and cathepsin B	TNV	Melanoma and colon cancer	[250]
	P2, pH and ROS	SRF/Ce6-loaded PEG-M-PPMT NPs	Lung cancer	[542]
RGD and EPR effects	Laser and GSH	RDG/shRNA	Breast cancer	[543]

**Fig. 7:**
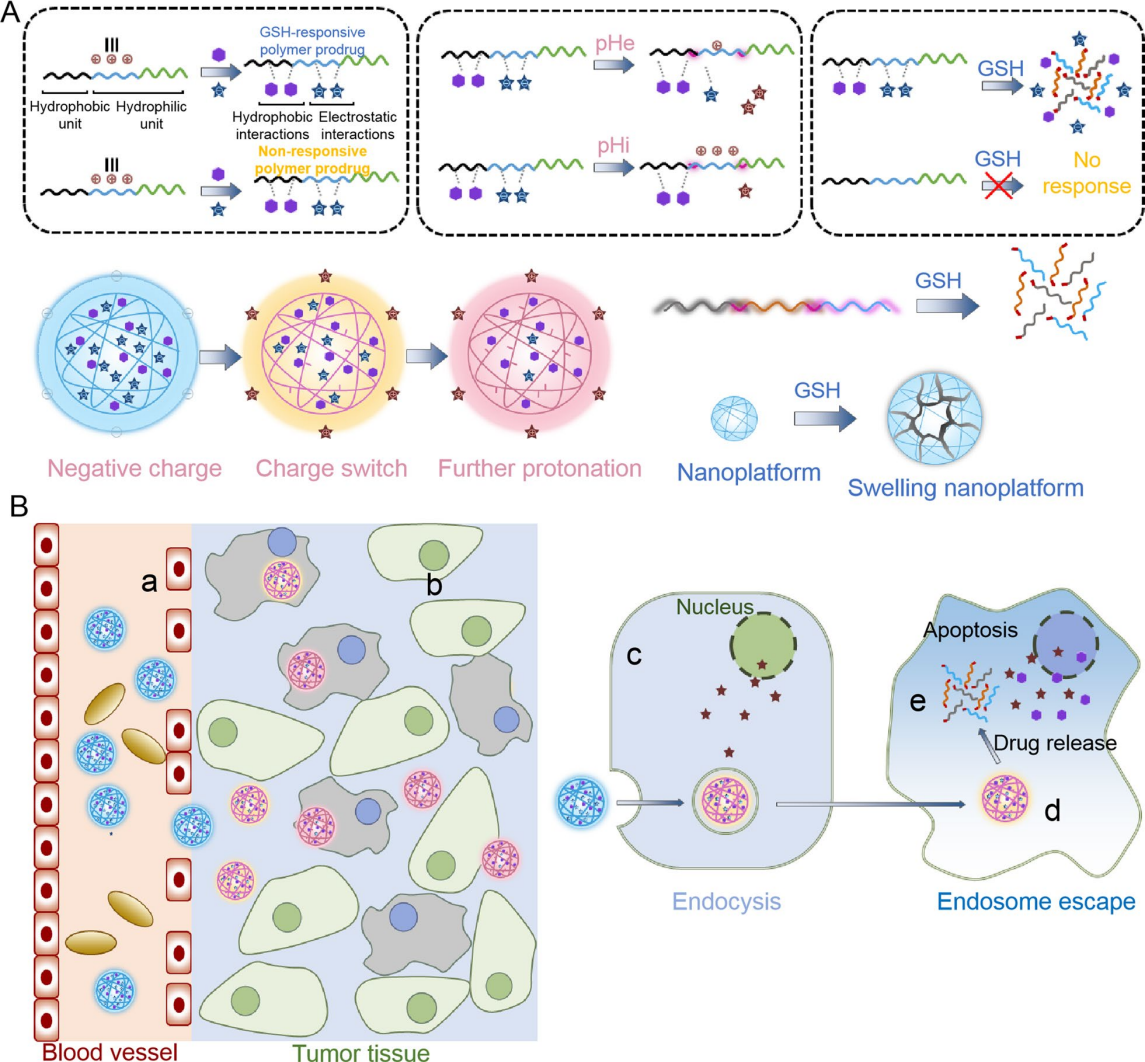
Schematic illustration of stimuli-responsive and targeted nanoplatforms for the specific delivery of therapeutic agents. **A** Preparation of the smart nanoplatform via electrostatic and hydrophobic interaction, and the pH-responsive surface charge switch, and GSH-responsive chemical degradation of polymer backbone. **B** Schematic illustration of and FA-mediated target and pH/GSH-responsive delivery processes: (a) the nanoplatforms show high stability in blood circulation; (b) therefore, they can effectively accumulate in tumor lesions via the EPR effect and receptor-mediated targeting; (c) acidic conditions can cause charge conversion of the nanoplatform; (d) endosome escape of the smart nanoplatform via proton-sponge effect; (e) intracellular GSH stimulation will trigger the release of therapeutic agents for tumor treatment

For example, a smart dual-responsive and targeting nanoplatform was prepared for the codelivery of chemotherapeutics (DOX and PTX) for treatment of lung adenocarcinoma [403] (Fig. 7). In this nanoplatform, FA was used as a receptor-mediated targeting molecule to facilitate the entry of these nanoplatforms into tumor cells. Moreover, acid-liable block copolymers and disulfide bonds endowed the nanoplatform with pH and GSH-responsive drug release behavior in the TME. It should be noted that the prepared nanoplatforms exhibited a surface charge switch from negative to positive during transmission from physiological environment to the TME, which can enhance tumor cells internalization. Subsequently, endosome escape of the nanoplatforms was achieved in the acidic endo/lysosome environment via the "proton-sponge" effect. As expected, this smart nanoplatform showed good biocompatibility, excellent cellular internalization, and improved tumor cell inhibition. Furthermore, the nanoplatform appeared synergistic and improved solid tumor killing efficiency compared with mono-chemotherapy in tumor-bearing mice models. This suggests that hybridization and combination of cancer nanomedicines present great promise for tumor treatment. In another important example, Jeong Hoon Byeon’s group [404] developed a platform for digitizable and continuous-flow manufacture in a compact and reconfigurable manner using a serial combination of plug-in reactionwares. This platform comprised three different composite nanocompounds with photothermally modulatable and structurally degradable characteristics for cancer treatment. As expected, these nanocompounds used for NIR-triggered chemothermal cancer therapy showed excellent anticancer efficacy with low side effects and effective renal excretion. Taken together, the hybridization and combination of nanomedicine appears to hold great promise for cancer treatment.

## Conclusions and prospects

In this review, we have focused on recent advances in receptor-mediated and stimuli-responsive active targeting strategies for cancer treatment. These versatile nanoparticles effectively overcome undirected drug biodistribution, undesired toxicity and high doses of administration, and play an important role in the development of novel chemotherapeutic agents and the understanding of their antitumor efficacy. Significant progress has been made in developing target-specific therapies leading to better cellular internalization and site-specific agent release by exploiting specific cancer cell surface receptors. These active targeting strategies not only enhance the efficacy of the drug but also reduce potential side effects. Additionally, stimuli-responsive targeting strategies with their unique characteristics have also shown high stability, enhanced tumor accumulation, and rapid release behaviors in response to exogenous or endogenous environmental stimuli both in vitro and in vivo. Taken together, precise delivery and specific release can be readily achieved by using the synergistic effects between versatile receptor-mediated and stimulus response targeting strategies, resulting in killing cancer cells within the tumor without damaging healthy tissues.

While the abovementioned approaches have many benefits, there are also some caveats. The size and surface characteristics of nanoplatforms can disrupt membranes and interfere with protein folding and membrane activity. These intracellular dysfunctions can further trigger feedback mechanisms such as “frustrated phagocytosis” [405]. Once administrated, the prepared nanoplatforms circulate in the bloodstream to access various tissues or organs. During this circulation, these nanoplatforms can interact with biomacromolecules (including carbohydrates, proteins, nucleic acids, and lipids) which can coat the nanoplatforms, leading to a surface or biomolecule corona, which alters the surface properties of the nanoplatforms, affects their therapeutic effects, and can induce protein unfolding [406–409]. Stimuli-responsive nanoplatforms are effective but can still be affected by their physiological environment. For example, most carbon nanomaterials (such as carbon nanoparticles and nanotubes) and metals (such as MnO) can act efficiently in acidic environments but generate ROS near tumors which can lead to cancer progression and metastasis [410, 411]. Notably, ROS-responsive nanoplatforms tend to be rapidly phagocytized due to their special surface properties [412, 413].

In the future, there is an urgent need to control the physicochemical features of nanoparticles to improve their targeting ability, especially their morphology, particle size distribution and surface chemistry. For example, new surface modification strategies need to be explored to confer novel multifunctionalities to the nanoparticles. Moreover, in order to improve the antitumor effects of nanoparticles the development of alternative reactions, formulations, or constructs containing stimulus components aimed at producing multiple strategies for highly effective combination cancer treatment should be a focus. Importantly, these new generation targeting strategies should be explored for an in-depth understanding of key parameters, such as their pharmacokinetics, biodistribution and nano-bio interfacial interactions, as such outcomes have a significant impact on cancer treatment. Furthermore, there are possibilities to develop novel stimuli-responsive modalities for better encapsulation of agents as well as their controlled release to further increase their therapeutic index with few side effects. It is forecast that nanoscale biomaterials comprising biocompatible lipids, polymers or inorganic materials in conjugation with targeting groups will have tremendous scope for transporting pharmaceutical active ingredients to their specific target sites for improved therapeutic purposes. Such versatile targeted nanoparticles will find broader application possibilities and will aid in the role out of personalized/precision medicine.

## Data Availability

Not applicable.

## Abbreviations

EPR: Enhanced permeability and retention
HA: Hyaluronic acid
TME: Tumor microenvironment
FRs: Folate receptors
Tf: Transferrin
FA: Folate acid
EGFRs: Epidermal growth factor receptors
HER2: Human epidermal growth factor receptor 2
DOX: Doxorubicin
PTX: Paclitaxel
TGF-α: Transforming growth factor-α
IFP: Interstitial fluid pressure
MOF: Metal organic framework
UIC: Ultrasound-mediated inertial cavitation
POxs: Poly(2-oxazo line)s
PMVE: Poly (methyl vinyl ether)
NMR: Nuclear magnetic resonance
GOx: Glucose oxidase
AZO: Azobenzene
Ce6: Chlorin e6
NQO1: NAD(P)H quinone dehydrogenase 1
GSH: Glutathione
PEG: Polyethylene glycol
CA IX: Carbonic anhydrase IX
Ab: Antibody
ATP: Adenosine triphosphate
TZ: Trastuzumab
PTT: Photothermal therapy
PDT: Photodynamic therapy
CDT: Chemodynamic therapy
RT: Radiotherapy
CAT: Catalase
ROS: Reactive oxygen species
RNS: Reactive nitrogen species
MR: Magnetic resonance
MMP: Matrix metalloproteinase
TaOx: Tantalum oxide
RBCs: Red blood cells
ICD: Immunogenic cell death
PNIPAAm: Poly-*N*-isopropylacrylamide
PNVCL: Poly(vinyl caprolactam)
PK: Protein kinase
ICP-OES: Inductively coupled plasma optical emission spectrometry
CD: Cluster of differentiation
ECM: Extracellular matrix
CPT: Camptothecin
DOTAP: 1,2-Dioleoyl-3-trimethylammonium-propane
AQ4N: Banoxantrone dihydrochloride
DTD: DT-diaphorase
TKIs: Tyrosine kinase inhibitors
